# The SlSHN2 transcription factor contributes to cuticle formation and epidermal patterning in tomato fruit

**DOI:** 10.1186/s43897-022-00035-y

**Published:** 2022-06-07

**Authors:** Cécile Bres, Johann Petit, Nicolas Reynoud, Lysiane Brocard, Didier Marion, Marc Lahaye, Bénédicte Bakan, Christophe Rothan

**Affiliations:** 1 UMR 1332 BFP, INRAE, Université de Bordeaux, 33140 Villenave d’Ornon, France; 2 Unité Biopolymères, Interactions, Assemblages, INRAE, BP71627, 44316 Nantes Cedex 3, France; 3 Univ. Bordeaux, CNRS, INSERM, Bordeaux Imaging Center, BIC, UMS 3420, US 4, 33000 Bordeaux, France; 4 INRA, UMR 1332 Biologie du Fruit Et Pathologie, 71 Av Edouard Bourlaux, 33140 Villenave d’Ornon, France

**Keywords:** Mutant, SHINE, Cuticle, Epidermis, Cell wall, Ethylene

## Abstract

Tomato (*Solanum lycopersicum*) is an established model for studying plant cuticle because of its thick cuticle covering and embedding the epidermal cells of the fruit. In this study, we screened an EMS mutant collection of the miniature tomato cultivar Micro-Tom for fruit cracking mutants and found a mutant displaying a glossy fruit phenotype. By using an established mapping-by-sequencing strategy, we identified the causal mutation in the *SlSHN2* transcription factor that is specifically expressed in outer epidermis of growing fruit. The point mutation in the *shn2* mutant introduces a K to N amino acid change in the highly conserved ‘mm’ domain of SHN proteins. The cuticle from *shn2* fruit showed a ~ fivefold reduction in cutin while abundance and composition of waxes were barely affected. In addition to alterations in cuticle thickness and properties, epidermal patterning and polysaccharide composition of the cuticle were changed. RNAseq analysis further highlighted the altered expression of hundreds of genes in the fruit exocarp of *shn2*, including genes associated with cuticle and cell wall formation, hormone signaling and response, and transcriptional regulation. In conclusion, we showed that a point mutation in the transcriptional regulator *SlSHN2* causes major changes in fruit cuticle formation and its coordination with epidermal patterning.

**Supplementary Information:**

The online version contains supplementary material available at 10.1186/s43897-022-00035-y.

## Core

In this study, we isolated the causal mutation underlying a tomato fruit glossy mutant by using an established mapping-by-sequencing strategy. A point mutation in the SlSHN2 transcriptional regulator was found to cause major changes in tomato fruit cuticle composition, architecture and properties and in epidermal patterning. The coordination by *SlSHN2* of the cutin and polysaccharides deposition in the fruit cuticle likely involves a cross-talk with various hormones, including ethylene.

## Gene & Accession

*SlSHN1* SGN accession: *Solyc03g116610*, *SlSHN2* SGN accession: *Solyc12g009490*, *SlSHN2_X1* NCBI accession: XP_004251719, *SlSHN2_X2* NCBI accession: XP_004251720, *SlSHN3* SGN accession: *Solyc06g053240*.

## Introduction

The plant cuticle is localized on the outer face of primary cell walls of epidermal cells of aerial plant organs where it serves as a protective barrier, notably by preventing water loss and resisting pathogen infection. In tomato fruit, which recently emerged as a model system for studying plant cuticle, the cuticle is thick and astomatous and influences commercially important traits such as visual appearance, susceptibility to fruit cracking and post-harvest shelf-life (Martin and Rose 2014; Petit et al. 2017). The cuticle is mainly composed of lipids, carbohydrates, and phenolics.

The surface of the fruit is covered by an epicuticular film of waxes. The wax fraction is typically a complex mixture composed of derivatives of very-long-chain fatty acids (VLCFA), mainly alkanes and alcohols, which may also include various secondary metabolites, such as amyrins, flavonoids and sterols. The synthesis of the aliphatic compounds involves several steps involving the synthesis of VLCFA through a multienzyme fatty acid elongase (FAE) complex and of VLCFA derivatives through either the alcohol forming pathway producing primary alcohols and wax esters, or the alkane forming pathway producing aldehydes, alkanes, secondary alcohols and ketones (Bernard and Joubès 2013). Export of waxes through plasma membrane occurs via ABC and LTPG transporters (Bernard and Joubès 2013).

The main component of the cuticle is cutin, which forms a continuum with the polysaccharides of the outer walls of epidermal cells. (Philippe et al. 2020a, 2020b). The cutin matrix is a polyester of polyhydroxy and epoxy fatty acids and fatty alcohols, with varying amounts of phenolic compounds (e.g. p-coumaric acid). In most plants, including tomato, the main cutin monomers are C16-based fatty acids (C16 dihydroxy fatty acids, C16 β-hydroxy fatty acids and C16 dicarboxylic acids) (Fich et al. 2016). The synthesis of cutin monomer starts with the synthesis of long chain fatty acids in the plastids. Fatty acids are transported to the cytoplasm where they undergo a series of modifications involving long-chain acyl-CoA synthetases (LACS), cytochrome P450-dependent fatty acid oxidases (CYP) and glycerol-3-phosphate acyl transferases (GPAT) to produce acyl-glycerols (Petit et al. 2016, 2017). Cutin precursors are then transferred via ABC transporters (Elejalde-Palmett et al. 2021) to the cell wall where they are assembled into a network of linear and branched cutin polymers (Philippe et al. 2016) by cutin synthases (Girard et al. 2012; Yeats et al. 2012; Fich et al. 2016).

In addition to the lipid polyesters, the roles of the polysaccharides and phenolic compounds in the structure and properties of the cuticle are increasingly being recognized (Philippe et al. 2020a, 2020b). The cuticle polysaccharides include pectins, which generally consist of polygalacturonans composed of the linear homogalacturonan (HG) and branched rhamnogalacturonan (RGI and RGII) (Atmodjo et al. 2013), hemicellulose that comprises xyloglucans and besides xylans and mananns (Lahaye et al. 2012), and cellulose (Jiang and Hsieh 2015). The cutin-embedded polysaccharides exhibit specific features including a high degree of esterification (i.e. methylation and acetylation) and a low ramification of the pectin RGI and a high crystallinity of cellulose (Philippe et al. 2020a). Together, cutin, cell wall polymers and phenolic compounds form the outer wall of the fruit and strongly contribute to the mechanical properties of the fruit skin (Philippe et al. 2020b; Khanal and Knoche 2017).

Here we used mapping-by-sequencing (MBS) to identify the causal mutation underlying a fruit cuticle mutant and established that a point mutation in the fruit-specific *SlSHN2* transcription factor is responsible for increased fruit glossiness and cuticle defects in tomato. Functional characterization of the mutant further showed that *shn2* mutation alone is sufficient to alter fruit cutin and cutin-embedded polysaccharides content, composition, degrees of esterification and polymerization, cuticle properties and epidermal patterning of the fruit. Remarkably, mutation of *SHN2* affected the expression of hundreds of genes in the fruit exocarp, among which those associated with lipid polyester biosynthesis, phenylpropanoid pathway, cell wall synthesis and modification, and more unexpectedly, hormone synthesis and signaling (mainly ethylene, auxins and gibberellins).

## Results

### Identification of the causal mutation underlying a fruit *glossy* mutant via mapping-by-sequencing

**Fig. 1:**
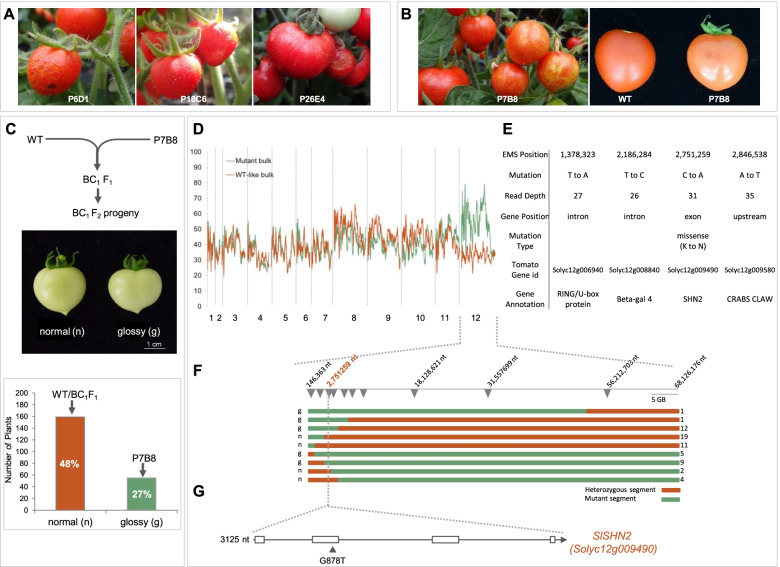
Mapping-by-sequencing identification of the *shn2* mutation responsible for the glossy fruit phenotype. **A** Examples of fruit cuticle alterations from three independent mutant families. **B** diversity of micro-cracks, glossiness and colour of red ripe fruits from P7B8 cuticle mutant. **C** The wild-type (WT) parental line was crossed with the P7B8 glossy mutant. The BC1F2 progeny (216 plants) was screened for glossy fruits (approximately 25% of the plants) and WT-like fruits (approximately 75% of the plants) to constitute the WT-like and the glossy bulks (38 individuals each). **D** Allelic frequencies (y axis) of glossy and WT-like bulks are represented along tomato chromosomes (x axis) by green and orange lines, respectively. A sliding window of 10 SNPs was used. E, High scoring mutations identified on chromosome 12. Only one mutation (position 2,751,259 on chromosome 12) located in exonic region has a possible deleterious effect on the protein. **F** Fine mapping of the causal mutation using the BC_1_F_2_ population. Recombinant analysis of 768 BC_1_F_2_ individuals allowed us to locate the causal mutation at position 2,751,259 nucleotides on SL3.0 Heinz 1706 tomato genome sequence. Marker positions are indicated by grey triangles. Number of recombinants are shown below the position of the markers. Chromosomal constitution of the recombinants are represented by green and orange bars, for mutant and heterozygous segments, respectively. G, the single nucleotide transversion G to T at position 2,751,259 in the second exon of *Solyc12g009490* leads to a missense mutation resulting in the K114N amino acid change

In previous studies, we isolated several *cutin-deficient* mutants (Shi et al. 2013; Petit et al. 2014, 2016) by screening an EMS-mutagenized tomato mutant population (Micro-Tom cultivar) (Just et al. 2013) for mutants showing increased fruit glossiness or colour changes. In the present study, we screened the same mutant population for mutants displaying more severe fruit surface defects including the presence of microcracks and evidences of wound-healing (Fig. 1A). We focused on one of these fruit surface mutants (line P7B8). The microcracks phenotype of P7B8 (Fig. 1B) was highly dependent on environmental conditions but consistently exhibited an enhanced fruit glossy phenotype (Fig. 1B). According to our previous study (Petit et al. 2014), this phenotype likely indicates a defect in cuticle formation and/or epidermal patterning. To facilitate identification of the mutant allele, we followed an established mapping-by-sequencing (MBS) strategy involving whole genome sequencing of bulked pools of individuals from a population segregating for the trait-of-interest (Garcia et al. 2016; Petit et al. 2016; Musseau et al., 2020). The homozygous P7B8 mutant line carrying a recessive *glossy* mutation was crossed with the WT (non-mutagenized) parental line in order to produce a BC_1_F_1_ hybrid plant, which was then selfed to generate a BC_1_F_2_ population (216 individuals). As the mutation underlying the microcracks/glossy phenotype is recessive, the BC_1_F_2_ population exhibited the expected phenotypic ratio of 3:1 between "normal" and "glossy" fruit phenotypes (Fig. 1C). DNAs from pools of individuals showing either phenotype (38 individuals per pool) were submitted for whole genome sequencing (tomato genome coverage depth of 32X-33X; Supplemental Table S1A) and trimmed sequences were mapped onto the reference tomato genome (Tomato Genome Consortium, 2012; SL3.0 Heinz 1706) (Supplemental Table S1B) to detect allelic variants.

Analysis of the allelic variant frequencies in the two bulks led to the identification of chromosome 12 as the genome region carrying the causal mutation, since it displayed low mutant allelic frequencies (AF < 0.4) in the WT-like bulk and much higher frequencies (AF > 0.95) in the glossy bulk (Fig. 1D and E and Supplemental Table S1C). Two chromosomal regions with high AF were detected on chromosome 12, which may be due to the presence of an unrelated introgression fragment from a wild tomato relative in the Micro-Tom cultivar, as described in Shirasawa et al. (2016). We therefore performed fine-mapping of the causal mutation using 768 BC_1_F_2_ individuals segregating for the glossy fruit phenotype and EMS-induced SNPs in the regions of interest as genetic markers (Fig. 1F). Recombinant analysis allowed us to locate the causal mutation in a single chromosomal region with high AF (Fig. 1F). Analysis of the putative effects on protein functionality of the four high scoring mutations found in this region highlighted only one mutation located in exonic region that is predicted to affect protein structure (Fig. 1E). This mutation at position 2,751,259 on chromosome 12 (SL3.0 Heinz 1706 tomato genome sequence) was unequivocally associated to the glossy mutant trait as demonstrated by further recombinant analysis (Fig. 1F and G). The causal mutation affected a predicted AP2/ERF transcription factor previously annotated SlSHN2 *(Solyc12g009490*; Shi et al., 2013) (Fig. 1G). SlSHN2 belongs to the SHINE clade of proteins which have an established function in the regulation of cuticle formation and epidermal patterning (Aharoni et al., 2004; Shi et al., 2011, 2013).

### The *shn2* mutation is located in the ‘mm’ conserved domain of SHN-like proteins

**Fig. 2:**
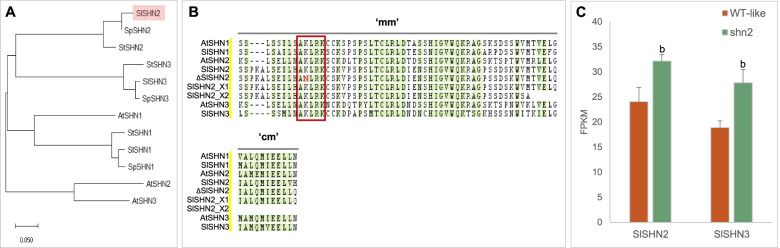
The amino acid mutated in SlSHN2 is located in a conserved middle motif of SHN. **A** Neighbour joining phylogenetic tree of SHN proteins from *Arabidopsis thaliana* (*At*), *Solanum lycopersicum* (*Sl*), *Solanum pennellii* (*Sp*) and *Solanum tuberosum* (*St*) species using MEGA11 (Tamura et al. 2021). AtSHN1, AT1G15360; AtSHN2, AT5G11190; AtSHN3, AT5G25390. SlSHN1, Solyc03g116610; SlSHN2, Solyc12g009490; SlSHN3, Solyc06g053240; SpSHN1, Sopen03g035520; SpSHN2, Sopen12g004490; SpSHN3, Sopen06g018310; StSHN1, Sotub03g030060; StSHN2, Sotub12g008480; StSHN3, Sotub06g017730. **B** Multiple sequence alignment of the characteristic conserved middle motif (termed ‘mm’) and C-terminal motif (termed ‘cm’) of SHN proteins using CLUSTALW (Larkin et al. 2007); 100% similar amino acids are indicated in pale green. The red box surrounds the conserved motif AKLRK. The mutated amino acid in the *shn2* mutant (ΔSHN2) is indicated in red. **C** Comparison of the expression of *SlSHN2* and *SlSHN3* genes in the WT-like and *shn2* mutant exocarp of 20 DPA fruit. Mean values of 3 biological replicates are given in FPKM with SD. b, *P* < 0.05 (Student’s *t* test)

Three *SHN*-like genes have been identified in the tomato genome (Shi et al. 2013). *SlSHN1 (Solyc03g116610*) appears to be closest at the amino acid level to *A. thaliana AtWIN1/SHN1* (Al-Abdallat et al. 2014) whereas *SlSHN3* (*Solyc06g053240)* is closest to *AtSHN3* (Shi et al. 2013). The SlSHN2 ortholog presents a lower homology with *A. thaliana* SHN proteins, ranging from 57 to 49% identity (66 to 63% similarity), and groups in a different cluster with other SHN2-like proteins from various Solanaceae species including the wild tomato relative *Solanum pennellii* and the potato *Solanum tuberosum* (Fig. 2A). While *SlSHN1* and *SlSHN3* genes contain a single intron, as the Arabidopsis *SHN*-like genes, *SlSHN2* contains a second intron. Furthermore, *SlSHN2* has a predicted splice variant that contains a third intron due to the alternative splicing of the second exon, thus indicating the possible production of two SlSHN2 isoforms. In addition to the AP2 domain, the isoform X1 (XP_004251719.1) shares with all SHNs from *A. thaliana* and other Solanaceae species the conserved middle and C-terminal domains, which are referred to as the 'mm' and 'cm' domains, respectively, in (Aharoni et al. 2004; Shi et al. 2011, 2013). The predicted isoform X2 (XP_004251720.1) also shares the conserved ‘mm’ domain, which is encoded by its third exon. However X2 (155 aa) lacks the ‘cm’ domain (Fig. 2B) and is therefore much shorter than X1 (220 aa). Recombinant analysis (Fig. 1F and G) clearly indicated that the single G to T nucleotide transition at the end of the second exon of *SlSHN2* is responsible for the glossy fruit phenotype of *shn2*. Specifically, the K114N mutation introduces a non-cationic amino acid change in the highly conserved cationic KLRK peptide motif of the ‘mm’ domain common to isoforms X1 and X2 (Fig. 2B).

In silico analysis of the expression of tomato S*HN*-like genes indicated that all three are weakly expressed in vegetative organs and predominantly expressed in reproductive organs (Shinozaki et al. 2018). *SlSHN2* is highly expressed in the young growing fruit, like *SlSHN3* which is also highly expressed in developing seeds (Supplementary Figure S1A). *SlSHN1* is highly expressed in unopened flower bud but not in the fruit. *SlSHN2* transcripts are abundant in the fruit outer epidermis in a narrow time window spanning the cell expansion phase, before the onset of ripening, from 10 days post anthesis (DPA) to Mature Green stage (MG) (Suppplementary Figure S1B). *SlSHN3* shares a similar pattern of expression in the fruit, being expressed from 5 DPA to MG stage. In contrast to *SlSHN2*, *SlSHN3* is equally expressed in the outer and inner epidermis (Supplementary Figure S1A, B and C). The transcript abundance of *SlSHN2* in fruit outer epidermis peaks at 20 DPA i.e. when fruit growth rate is maximum (Guillet et al. 2002) at which stage it is twice higher than that of *SlSHN3* (Supplementary Figure S1B).

To analyze the expression of differentially expressed genes (DEGs), we performed RNAseq analysis of 20DPA fruit exocarp from *shn2* and WT. The WT control plants used for RNAseq analysis, named WT-like, are BC_1_F_2_ individuals which do not carry the *shn2* mutation. RNAseq analysis indicated that *SlSHN2* and *SlSHN3* were both detected in 20 DPA fruit exocarp (Fig. 2C) of WT and *shn2*. Interestingly, transcript abundances of *SlSHN2* and *SlSHN3* were higher in the *shn2* mutant (significant at *P* < 0.05 but not at *P* < 0.01) indicating that the *shn2* mutation has no deleterious effect on transcript stability of *SlSHN2*. Mapping of the reads onto the tomato genome (ITAG3.2) confirmed the presence of *SlSHN2* X1 and X2 transcripts variants (Supplementary Figure S2). Counting reads further indicated that the ratios of X1 and X2 transcripts are similar in *shn2* and WT. As expected, *SlSHN1* was not detected.

### Fruit from the *shn2* mutant shows severe cutin-deficiency

**Fig. 3:**
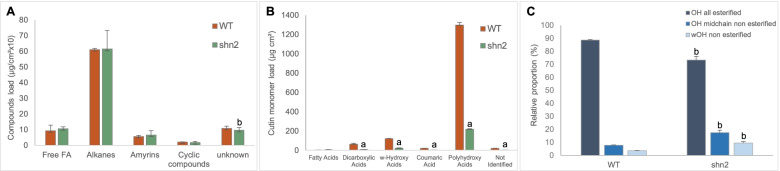
Fruit from the *shn2* mutant shows severe cutin-deficiency. **A** Wax load and composition of the fruit cuticle from wild-type (WT) and *shn2* plants. Values (µg/cm^2^ × 10) are mean ± SD (*n* = 3). **B** Cutin load and composition of fruit cuticle from wild-type (WT) and *shn2* plants. Values (µg/cm^2^) are mean ± SD (*n* = 3). **C** Esterification degree of the 9(10),16-dihydroxyhexadecanoic acid OH groups of tomato fruit cutin. Values are mean ± SD (*n* = 3). a, *P* < 0.01; b, *P* < 0.05 (Student’s *t* test)

In order to investigate how the mutation in *SlSHN2* gene affects cuticle composition, we analyzed the wax and cutin constituents of 20 DPA fruit cuticle. No differences in wax loading were observed for the major classes of compounds between WT and *shn2* fruits (Fig. 3A). However, detailed analysis of wax compounds revealed differences in alkane composition (Supplementary Table S2), including decreased levels in C25, C27 (2.9-fold reduction) and increased levels in C33 (1.6-fold increase) alkanes in the *shn2* mutant. These variations were likely dependent on the decarboxylation pathway of alkane biosynthesis and not on the reductive pathway because only odd-numbered alkanes were significantly affected (Bernard and Joubès 2013).

In contrast to waxes, the total cutin load in *shn2* was reduced by 5.5-fold, from 1674 to 302 µg/cm^2^ (Supplementary Table S3); this reduction affected all classes of compounds (Fig. 3B). This corresponded to a ~ sixfold drop in the amount of the major cutin monomer 9(10), 16-dihydroxyhexadecanoic acid, a 7 to ~ 12-fold reduction of dicarboxylic acids C16:0 DCA and C16:0 DCA 9(10) OH and a 3.3 to 6.3-fold reduction of ɷ-hydroxy acids C16:0 ɷOH, C16:0 ɷOH 10-oxo, C18:0 ɷOH (9 or 10) OH and C18:0 ɷOH (9, 10) epoxy. Cutin reduction was not restricted to fatty acids-related monomers as the phenylalanine-derived *p*-coumaric acid was also severely reduced (tenfold reduction). As cuticle function is closely related to the structure of the cutin polymer, in which cutin polymerization plays a key role, we further checked the esterification degree of the OH groups from 9(10), 16-dihydroxyhexadecanoic acid using an established technology (Philippe et al. 2016). All esterified OH were significantly reduced in *shn2* while midchain and ɷnon-esterified OH were increased by more than two-fold (Fig. 3C).

### Cuticle thickness and properties are altered in the *shn2* mutant

**Fig. 4:**
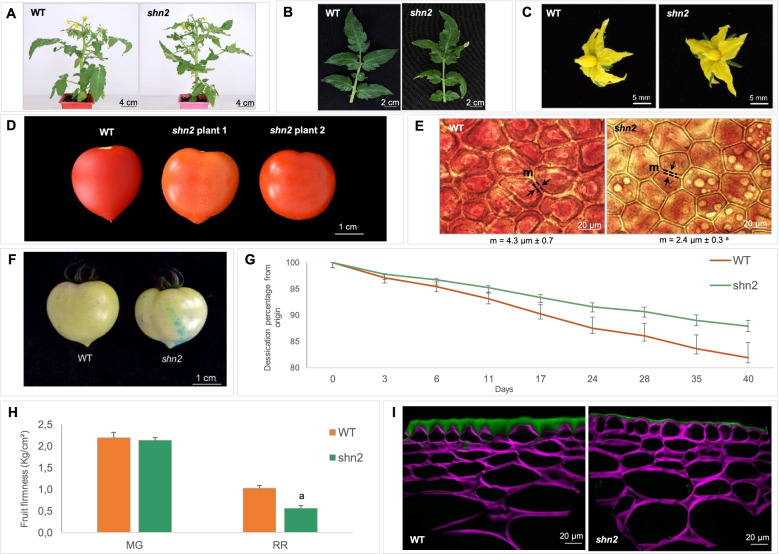
Comparison of the developmental, cuticle and epidermal phenotypes of *shn2* mutant and wild-type (WT). **A**-**C** The *shn2* mutation has no effect on the architecture of 6 weeks-old plants (**A**) and on the morphology of the fourth leaf (**B**) and flower at anthesis (**C**). **D** Variability in fruit colour and glossiness of *shn2* fruits. **E** Microscopic observations of freshly peeled outer epidermis of red ripe (RR) fruit. The white bodies within the cells are likely oil bodies. The width of the cutinized cell wall was measured between the two black arrowheads (m). Mean values (in µm) of 100 measures (10 measures on 10 sections) are given with SD. a, *P* < 0.01 (Student’s *t* test). **F** Macroscopic observations of cuticle permeability of mature green (MG) fruits after overnight soaking in 0.1% toluidine blue. **G** Water loss progression in RR fruit was measured over 40 d of postharvest storage at room temperature. (*n* = 5). **H** Fruit firmness of MG and RR fruits. Values are mean ± SD of 3 measures on 6 fruits. a, *P* < 0.01 (Student’s *t* test). **I** Confocal fluorescence microscopy of exocarp cell layers using Calcofluor and Bodipy dual staining showing reduced cuticle deposition and loss of conical epidermal cell shape in the *shn2* fruit. Scale bars are indicated on each pictures. MG, mature green; RR, red ripe

Consistent with the strong and preferential expression of *SlSHN2* in young developing fruit, whole plant, leaf and flower phenotypes were identical in *shn2* and WT (Fig. 4A, B and C). Mutant fruits were consistently glossier and the ripe fruits had an orange hue (Fig. 4D). The expressivity of both traits was however variable (Fig. 4D) and *shn2* fruits were more or less glossy or orange according to environmental factors such as growth season and position of the plant in the greenhouse. In agreement with the strongly reduced cutin content (Supplementary Table S3), the most striking effect of the *shn2* mutation was on the fruit cuticle. The width of the anticlinal cutinized cell wall between adjacent epidermal cells was substantially reduced, by almost two-fold, in the *shn2* mutant (Fig. 4E). In addition, depending on the environmental conditions, subcellular structures reminiscent of oil bodies could be observed in epidermal cells of ripe *shn2* mutant fruits (Fig. 4E). These profound changes in fruit cuticle led to modifications in fruit cuticle permeability as measured by toluidine blue staining (Fig. 4F). Unexpectedly, water loss was not increased but greatly reduced in *shn2* (Fig. 4G). Moreover, fruit firmness measured at red ripe stage with a penetrometer was also significantly reduced in *shn2* (Fig. 4H). As this may result from alterations in cuticle properties and epidermal cell features, we further analyzed the characteristics of epidermal and sub-epidermal cells of 20 DPA tomato fruit by BODIPY staining of neutral lipids and Calcofluor-white staining of cellulose (Fig. 4I). The results revealed a considerably thinner cuticle in *shn2* than in WT, the absence of cuticular anticlinal pegs between epidermis cells and the lack of cutinization of sub-epidermal cell layers in *shn2*. Moreover, we observed that epidermal cells from *shn2* did not display the characteristic cone-shaped morphology of WT cells but were round-shaped with more intense staining of epidermal cell walls. No obvious variations in cell size or morphology were observed in sub-epidermal cells. The same observations were made in WT-like plants (Supplementary Figure S3) indicating that the phenotypic alterations observed were independent from other EMS mutations found in *shn2*. Taken together, these results indicate that the *shn2* mutation profoundly alters both cuticle deposition and epidermal patterning.

### Composition of cutin-embedded polysaccharides is significantly altered in the *shn2* mutant

**Fig. 5:**
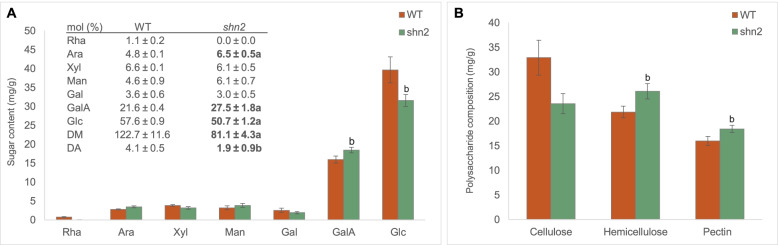
Cuticle-embedded polysaccharides are altered in the *shn2* mutant. **A** Sugar content; the inset table represents the sugar composition expressed as total sugar mole percentage. The degree of methyl esterification (DM) refers to the molar amount of methanol per 100 mol of galacturonic acid while the degree of acetylation (DA) is reported as the weight of acetic acid/weight of polysaccharides. Values are mean ± SD (*n* = 3). Values in bold indicate significant difference from the WT. a, *P* < 0.01; b, *P* < 0.05 (Student’s *t* test). Rha, rhamnose; Ara, arabinose; Xyl, xylose; Man, mannose; Gal, galactose; GalA, galacturonic acid; Glc, glucose. **B** Polysaccharide composition. Cellulose: glucose deduced from second hydrolysis; hemicellulose: total neutral sugar with glucose from second hydrolysis only; pectin: galacturonic acid. Values are mean ± SD (*n* = 3)

To further investigate the impact of *shn2* mutation on epidermal patterning, we analyzed the composition of cutin-embedded polysaccharides (CEP) (Philippe et al. 2020a) (Fig. 5). Significant differences were observed between the *shn2* mutant and WT regarding the galacturonic acid content, which was 1.15-fold higher in *shn2*, and in glucose, which was 1.26-fold lower in *shn2* (Fig. 5A). Because variations in sugar composition likely reflect changes in the abundance of the various cell wall polymers, we next analyzed the composition of CEP (Fig. 5B). *shn2* displayed a significant decrease in cellulose (1.4-fold) and, in contrast, significant increases in both hemicellulose (1.19-fold) and pectin (1.15-fold). To get further insight into polysaccharide structure, we examined the molar ratios of the various sugars present in CEP (Fig. 5A inset). The ratio of ((Ara + Gal)/Rha) is currently used to evaluate the extent of rhamnogalacturonan (RGI) branching while the ratio (uronic acid)/(rhamnose + arabinose + galactose) is used to evaluate the linearity of pectins embedded within the cutin layers (Houben et al. 2011; Philippe et al. 2020a). The ratio of (Xyl/Man) is used to evaluate the proportion of mannan to xyloglucane in the hemicellulose fraction. These ratios showed a higher branching level of RGI in WT fruit than in *shn2*, where rhamnose content was below the detection limit of the method. A higher proportion of mannan in the hemicellulose fraction of *shn2* was observed compared to WT. This was accompanied by a decrease in pectin methylesterification (X 1.5 fold) and a considerable decrease in the acetylation of CEP (X 2.2 fold) (Fig. 5A inset).

### Analysis of differentially expressed genes in developing fruit exocarp of *shn2* and WT

Since the *shn2* mutation has a pleotropic effect on fruit epidermis and affects not only cuticle formation but also epidermal cell wall and morphology, we further investigated gene expression changes in the fruit exocarp. The fruit exocarp is defined here as the tissue obtained during the peeling of the fruit; it is mostly constituted of epidermal cells and underlying cutinized sub-epidermal cells (Petit et al. 2016; Renaudin et al. 2017). The 20 DPA developmental stage was selected because it corresponds to the maximum rate of fruit growth (Guillet et al. 2002) and cuticle formation in Micro-Tom (Mintz-Oron et al. 2008; Petit et al. 2014), and to the peak of *SlSHN2* expression in the fruit (Supplemental Figure S1). In order to exclude any potential effect of additional EMS mutations carried by the P7B8 line on gene expression changes in the mutant and to take into account the variable expressivity of the *shn2* mutation, we constituted pools of exocarp *shn2* and WT-like samples as follows. After recombinant analysis, BC1F3 homozygous progenies for either the mutant allele (‘*shn2*/*shn2*’ genotype; hereafter referred to as *shn2*) or the WT allele (‘SHN2/SHN2’ genotype; hereafter referred to as WT-like) were used to collect 20 DPA fruit exocarp.

Transcripts expressed in the fruit exocarp were subjected to high-throughput RNA sequencing analysis (RNAseq) using Illumina technology. Among the ~ 21,891 genes detected, 1927 were found to be differentially expressed between WT-like and *shn2* fruit exocarp using a two-fold cut-off on fold change in transcript abundance (ratio of 1 and -1 on a log2 scale) and a *q*-value < 0.05. Of these, 1433 genes and 494 genes were expressed at higher or lower levels, respectively, in *shn2* than in WT-like. We further restricted our analysis to the most highly expressed genes by using an additional cut-off on the normalized number of reads (FPKM > 5 in either the mutant or the WT-like). This stringent analysis selected 921 genes among which the majority (753) was expressed at higher levels while 168 DEGs were expressed at lower levels in the *shn2* mutant (Supplementary Figure S4A). A wide range of biological processes were identified within the DEGs by Gene Ontology (GO) term enrichment analysis (Supplementary Figure S4B). Several categories related to the phenotype-of-interest, including cuticle formation and regulation, cell wall synthesis and modification, regulation of developmental and defense processes, and hormone-associated processes were expressed at higher or lower levels in the *shn2* mutant. The largest categories comprised DEGs associated with the regulation of transcription (78 DEGs) (Supplemental Table S4) and DEGS related to plant defense. As expected, significant differences were observed for lipid-related genes including 29 DEGs associated with cuticle formation (Table 1). We also detected 50 DEGs possibly involved in cell wall formation and modifications (Table 1 and Supplemental Table S5), in agreement with previous studies of lines in which the expression of *SHINE* was altered (Shi et al. 2011, 2013) and of tomato *gpa6a* mutant (Petit et al. 2016). Notably, we observed a number of DEGs (58) involved in hormone biosynthesis, signaling and action (Table 2). Among these were 29 DEGs associated with the ethylene-activated signaling pathway, one of the biological process ranking amongst the top 10 gene ontology terms (Supplementary Figure S4B). Several DEGs likely associated with the control of epidermal differentiation and development were also detected (Supplemental Table S6).

**Table 1:** Differentially expressed genes (DEGs) associated to cuticle modifications and cell wall polysaccharides

Category	Gene Identification	Putative Function	Log2 Fold Change *shn2*/WT	*q* value
**Wax Biosynthesis**	Solyc05g013220	Ketoacyl-CoA synthase KCS	-1.97	5.0E-62
	Solyc06g074390	Fatty acyl-CoA reductase FAR1	1.24	3.5E-17
	Solyc03g117800	ECERIFERUM 3 CER3	-1.28	6.4E-58
	Solyc10g080840	Cytochrome P450 MAH1	-1.86	8.6E-209
**Cutin Biosynthesis**	Solyc08g081220	Cytochrome P450 CYP86A69 CD3	-2.20	1.3E-254
	Solyc04g082380	Cytochrome P450 CYP86A8-like	-1.10	4.4E-08
	Solyc09g014350	Glycerol-3-phosphate acyltransferase GPAT6	-1.20	8.9E-69
**Lipid Transport**	Solyc10g075100	Non-specific lipid-transfer protein LTP2_1e	2.98	0.0E + 00
	Solyc09g018010	Non-specific lipid-transfer protein LTP2_2	1.37	6.9E-16
	Solyc10g075110	Non-specific lipid-transfer protein LTP2_1f	2.50	8.4E-31
	Solyc08g067540	Non-specific lipid-transfer protein	1.57	3.4E-07
	Solyc05g015490	Plant lipid transfer protein LTPG1	-3.37	7.0E-88
**Polymerization**	Solyc02g071610	GDSL esterase/lipase	1.58	7.3E-93
	Solyc02g071700	GDSL esterase/lipase	2.41	2.1E-50
	Solyc07g049440	GDSL esterase/lipase	-1.88	0.0E + 00
	Solyc03g121180	GDSL esterase/lipase	1.08	6.8E-74
	Solyc10g076740	GDSL esterase/lipase CPRD49	-1.10	8.9E-29
	Solyc11g006250	GDSL esterase/lipase CUS1	1.22	1.4E-69
	Solyc06g083650	GDSL esterase/lipase CUS4	-1.12	1.1E-33
	Solyc08g008610	Alpha/beta-Hydrolases superfamily protein BDG1	3.53	7.1E-151
**Regulation**	Solyc02g088190	SlMIXTA-like	-1.08	1.0E-59
**Phenylpropanoid Pathway**	Solyc05g056170	Phenylalanine ammonia-lyase	-1.84	1.6E-122
	Solyc09g007890	Phenylalanine ammonia-lyase	1.17	4.8E-87
	Solyc10g086180	Phenylalanine ammonia-lyase	1.57	3.3E-42
	Solyc09g007910	Phenylalanine ammonia-lyase	2.47	2.8E-202
	Solyc00g500353	Phenylalanine ammonia-lyase	4.59	0.0E + 00
	Solyc09g007920	Phenylalanine ammonia-lyase	1.08	7.8E-84
	Solyc02g093230	Caffeoyl-CoA O-methyltransferase	1.78	9.9E-34
	Solyc02g093250	Caffeoyl-CoA O-methyltransferase	1.79	2.9E-50
	Solyc09g091510	Chalcone synthase 1 CHS1	1.91	6.6E-121
	Solyc01g111070	Chalcone synthase-like	1.32	3.6E-30
**Cell Wall**	Solyc12g015770	Cellulose synthase	-2.00	3.7E-83
	Solyc07g043390	Cellulose synthase	1.67	5.3E-41
	Solyc03g097050	Cellulose synthase	3.96	0.0E + 00
	Solyc02g089640	Cellulose-synthase-like C4	1.71	1.8E-189
	Solyc01g065530	COBRA-like protein	1.92	2.2E-221
	Solyc08g081620	Endo-1,4-beta-glucanase precursor	-1.05	1.9E-07
	Solyc08g082250	Endo-beta-1,4-D-glucanase Cel8	1.01	7.5E-88
	Solyc07g049300	Endoglucanase	1.18	1.5E-61
	Solyc07g064870	Endoglucanase	1.59	5.1E-71
	Solyc03g071570	Pectate lyase	1.29	3.0E-60
	Solyc09g091430	Pectate lyase	1.90	9.2E-102
	Solyc09g061890	Pectate lyase	2.26	5.4E-231
	Solyc04g082140	Pectinesterase	1.31	4.6E-116
	Solyc06g009190	Pectinesterase	1.20	7.4E-36
	Solyc03g123620	Pectinesterase	2.71	0.0E + 00
	Solyc03g083730	Plant invertase/pectin methylesterase inhibitor	1.65	2.8E-12
	Solyc12g010540	UDP-glucuronate 4-epimerase 4	1.70	3.9E-135
	Solyc05g051260	1,4-beta-xylanase	2.40	8.5E-07
	Solyc03g093080	Xyloglucan endotransglucosylase/hydrolase	3.50	0.0E + 00
	Solyc07g055990	Xyloglucan endotransglucosylase/hydrolase	4.05	4.4E-208
	Solyc02g080160	Xyloglucan endotransglucosylase/hydrolase	1.62	3.6E-101
	Solyc07g056000	Xyloglucan endotransglucosylase/hydrolase	3.09	0.0E + 00
	Solyc03g093110	Xyloglucan endotransglucosylase/hydrolase	3.96	0.0E + 00
	Solyc07g009380	Xyloglucan endotransglucosylase-hydrolase 2	-1.14	3.0E-86
	Solyc03g093130	Xyloglucan endotransglucosylase-hydrolase 3	4.19	0.0E + 00
	Solyc12g011030	Xyloglucan endotransglucosylase-hydrolase 9	3.02	5.7E-234
	Solyc12g017240	Xyloglucan endo-transglycosylase B1	2.73	0.0E + 00
	Solyc07g044960	Xyloglucan galactosyltransferase KATAMARI1	3.89	6.7E-72
	Solyc03g093120	Xyloglucan:xyloglucosyl transferase TCH4	4.87	0.0E + 00
	Solyc06g074670	UDP-apiose/UDP-xylose synthase	2.57	0.0E + 00

DEGs in the exocarp of WT-like and *shn2* 20 DPA fruit. Genes were assigned manually to functional categories. Annotations are from SGN

**Table 2:** Differentially expressed genes (DEGs) associated to hormones

Category	Gene Identification	Putative Function	Log2 Fold Change *shn2*/WT	*q* value
Gibberellin	Solyc12g006460	GA ent-kaurenoate oxidase	4.26	2.5E-249
	Solyc07g056670	Gibberellin 2-oxidase	-3.52	4.2E-46
	Solyc09g074270	Gibberellin receptor	2.34	1.1E-87
	Solyc04g017720	Gibberellin regulated protein GASA6	-2.04	2.3E-17
	Solyc04g078195	Gibberellin-regulated protein	1.12	5.0E-84
Ethylene synthesis and signalling	Solyc07g049550	1-aminocyclopropane-1-carboxylate oxidase 2 ACO2	-3.35	3.8E-26
	Solyc07g026650	1-aminocyclopropane-1-carboxylate oxidase 5 ACO5	-1.47	2.3E-13
	Solyc02g091990	Aminocyclopropane-1-carboxylate synthase 3 ACS3	3.48	1.4E-74
	Solyc09g089610	Ethylene receptor-like protein ETR6	-1.81	5.3E-38
Ethylene response	Solyc10g006130	EAR motif SlERF36	1.30	3.1E-135
	Solyc05g052030	Ethylene response factor 4	-3.90	8.6E-42
	Solyc03g093610	Ethylene response factor A.2	2.42	3.0E-144
	Solyc04g014530	Ethylene response factor C.2	-1.08	7.0E-14
	Solyc02g077370	Ethylene response factor C.5	1.74	2.1E-29
	Solyc03g093560	Ethylene response factor C.6	2.32	2.5E-35
	Solyc01g108240	Ethylene response factor D.3	3.96	0.0E + 00
	Solyc10g050970	Ethylene response factor D.4	4.26	0.0E + 00
	Solyc07g053740	Ethylene response factor F.4	1.29	2.1E-20
	Solyc05g051200	Ethylene-responsive factor 1	-2.02	6.8E-31
	Solyc04g011440	Ethylene-responsive heat shock protein cognate 70	2.44	0.0E + 00
	Solyc02g070040	Ethylene-responsive nuclear protein ERT2	4.10	3.8E-135
	Solyc08g080630	Ethylene-responsive proteinase inhibitor 1	-7.90	4.6E-25
	Solyc12g056980	Ethylene-responsive transcription factor	-1.18	4.6E-15
	Solyc11g012980	Ethylene-responsive transcription factor	1.52	1.2E-62
	Solyc06g035700	Ethylene-responsive transcription factor	3.60	5.2E-59
	Solyc11g042560	Ethylene-responsive transcription factor	5.27	3.6E-56
	Solyc08g082210	Ethylene-responsive transcription factor	-1.43	2.3E-43
	Solyc12g009240	Ethylene-responsive transcription factor	1.26	3.2E-107
	Solyc01g090560	Ethylene-responsive transcription factor	1.60	3.3E-30
	Solyc01g090340	Ethylene-responsive transcription factor 2	3.88	3.5E-77
	Solyc02g077840	Ethylene-responsive transcription factor 4	1.17	1.1E-18
	Solyc08g078190	Ethylene-responsive transcription factor 5	1.15	1.7E-32
	Solyc03g093550	Ethylene-responsive transcription factor 5	1.62	4.6E-72
	Solyc03g093540	Ethylene-responsive transcription factor 5	2.28	6.1E-12
	Solyc04g078640	Ethylene-responsive transcription factor RAP2-1	-1.85	8.0E-11
Auxin synthesis, transport and response	Solyc06g062920	Auxin-regulated dual specificity cytosolic kinase	2.57	4.1E-236
	Solyc03g120380	Auxin-regulated IAA19	1.51	2.0E-37
	Solyc06g008580	Auxin-regulated IAA22	1.69	2.4E-22
	Solyc08g021820	Auxin-regulated IAA29	-2.31	5.2E-31
	Solyc12g096570	Auxin-regulated organ size gene	-4.27	1.6E-31
	Solyc06g075690	Auxin-regulated protein AF416289	4.87	5.5E-203
	Solyc04g053010	Auxin-responsive protein SAUR65	1.56	3.4E-74
	Solyc01g099840	Dormancy/auxin associated protein	-1.15	1.3E-44
	Solyc10g079640	IAA-amino acid hydrolase ILR1-like 6	1.74	2.1E-76
	Solyc06g059730	PIN6	6.11	3.3E-51
	Solyc01g110920	Small auxin up-regulated RNA26	-1.06	2.0E-18
	Solyc04g081250	Small auxin up-regulated RNA51	-1.39	2.4E-17
	Solyc04g081270	Small auxin up-regulated RNA52	-1.93	8.6E-24
Abscisic Acid	Solyc02g084850	Abscisic acid environmental stress-inducible protein TAS14	1.13	3.8E-08
	Solyc03g095780	Abscisic acid receptor PYL4-like	1.99	3.7E-48
	Solyc08g005610	Cytochrome P450 CYP707A ABA	3.18	4.3E-26
Brassinosteroid	Solyc02g071990	Brassinosteroid signaling positive regulator-related protein BES1/BZR1	1.20	1.9E-55
	Solyc07g062260	Brassinosteroid signaling positive regulator-related protein BES1/BZR1	1.55	6.4E-34
Cytokinin	Solyc11g069570	Cytokinin riboside 5'-monophosphate phosphoribohydrolase	1.26	8.0E-12
Jasmonate	Solyc09g061840	3-ketoacyl-CoA thiolase peroxisomal-like	1.02	2.8E-26
	Solyc07g006890	Cytochrome P450 CYP94B jasmonate	3.01	0.0E + 00
	Solyc12g009220	Jasmonate ZIM-domain protein 1	6.33	2.1E-139
	Solyc07g042170	Jasmonate ZIM-domain protein 3	1.82	3.9E-187
	Solyc08g076930	Jasmonic acid 3	1.11	3.2E-76

DEGs in the exocarp of WT-like and *shn2* 20 DPA fruit. Genes were assigned manually to functional categories. Annotations are from SGN

### Genes involved in cuticle formation

Of the 29 DEGs involved in cuticle formation, 4 genes were associated with wax biosynthesis, 12 with cutin biosynthesis and assembly, 5 with lipid transport and 10 with phenylpropanoid pathway (Table 1). All wax-related genes were expressed at lower levels in the *shn2* mutant, with the exception of *Solyc06g074390*. While the expression of the *KCS/CER6* gene *(KCS_23; Solyc05g013220.2)* encoding a β-ketoacyl-CoA synthase enzyme that catalyzes the first step in the multi-enzymatic fatty acid elongase complex generating very long chains (VLCs) C20-C36 acyl-CoAs was lower in *shn2*, the expression of the *FAR/CER4* gene (*FAR_1a*; *Solyc06g074390*) encoding a fatty acyl-CoA reductase enzyme involved in the reductive pathway producing even-numbered carbon primary alcohols from VLCs-acyl-coAs was higher. At the opposite, the expression of two genes from the decarboxylation pathway yielding odd-numbered alkanes from VLC acyl-coAs was lower in the *shn2* mutant. CER3 (*Solyc03g117800*) likely catalyzes the conversion of VLC acyl-CoAs to intermediate VLC aldehydes, and in association with CER1, to VLC alkanes. The expression of *CER3* is also strongly reduced in the *SlMIXTA-like* RNAi lines (Lashbrooke et al. 2015). MAH1 (CYP96A15, *Solyc10g080840*) is a midchain alkane hydroxylase responsible for the generation of secondary alcohols and ketones derivatives from alkanes (Bernard and Joubès 2013). The cutin-related genes displayed contrasted behaviors in the *shn2* mutant compared with the WT. Cutin biosynthesis genes expressed at lower levels in *shn2* encode enzymes known to be required for the production of the MAG cutin precursors in tomato. They include two cytochrome P450-dependent fatty acid oxidases (CYP) for fatty acid ɷ-hydroxylation (*CYP86A69, Solyc08g081220; CYP86A8-like*, *Solyc04g082380)* (Shi et al. 2013) and one glycerol-3-phosphate acyltransferase (*GPAT6, Solyc09g014350*) (Petit et al. 2016) (Table 1). Notably, both *CYP86A69* and *CYP86A8-like* have been demonstrated to be SlSHN3 target genes (Shi et al. 2013). Five DEGs encoded nonspecific lipid transfer (nsLTP) protein, examples of which have been suggested to be involved in the transport of cuticle components across the apoplast, although this has yet to be conclusively demonstrated (Yeats and Rose 2008). All were expressed at higher levels excepted for the *Solyc05g015490* gene encoding LTPG1, which was expressed at lower level. LTPG1, which is a major nsLTP from tomato fruit cuticle and a known allergen (Le et al. 2006), is also expressed at lower level in *cus1* RNAi lines (Girard et al. 2012) and in *gpat6a* tomato mutant (Petit et al. 2016). Seven GDSL-lipase genes were oppositely expressed at lower levels (3 DEGs) or at higher levels (4 DEGs). The *Solyc10g076740* gene encoding the CUS1 enzyme that catalyzes the extracellular polymerization of cutin (Girard et al., 2012; Yeats et al., 2012) and is associated with the growth of the outer epidermal wall (Segado et al. 2020) was expressed at higher level, in contrast to its fate in the *gpat6a* tomato mutant (Petit et al. 2016). In contrast, the gene encoding CUS4 (*Solyc06g083650*), which displays the same developmental pattern as CUS1 during early fruit development but is much less expressed (Segado et al. 2020), was expressed at lower level in *shn2*. The α/β-hydrolase BODYGUARD1 (*BDG1*; *Solyc08g008610*) is orthologous to the Arabidopsis AtBDG that was shown to be required for cutin biosynthesis, possibly through cutin polymerization (Jakobson et al. 2016). At last, a *SlMIXTA-like* gene (*Solyc02g088190*) encoding a MYB transcription factor at the core of the regulation of cutin biosynthesis in land plants (Xu et al. 2020) was expressed at lower level. Silencing of the epidermis-expressed *SlMIXTA-like* gene in tomato results in the alteration of flattening of fruit epidermal cells, reduction in cuticle thickness and reduced expression of numerous cutin biosynthesis and assembly genes (Lashbrooke et al. 2015), including the GDSL-lipase genes *Solyc07g049440* and *Solyc03g121180* which were also less expressed in *shn2*.

The RNAseq data also indicated that the phenylpropanoid and flavonoid biosynthetic pathways were altered in *shn2* (Table 1). In the phenylpropanoid core pathway, five phenylalanine ammonia-lyase (PAL) genes (*Solyc00g300353, Solyc09g007890, Solyc09g007910, Solyc09g007920* and *Solyc10g086180*) were expressed at higher levels in *shn2* while only one (*Solyc05g056170)* was expressed at lower level. PAL catalyzes a committed step in the phenylpropanoid pathway that leads, via the *p*-coumaric acid that accumulates in the cuticle during fruit ripening (Lara et al. 2015), to the synthesis of *p*-coumaroyl-CoA that is a precursor of caffeoyl-CoA. Two caffeoyl-CoA O-methyltransferases (CCOAOMT, *Solyc02g093230* and *Solyc02g093250*) catalyzing the formation of feruloyl-CoA from caffeoyl-CoA were up-regulated in *shn2*. In the flavonoid biosynthetic pathway, transcripts levels of two chalcone synthases (CHS1, *Solyc09g091510* and CHS-like *Solyc01g111070*) were higher in *shn2*. CHS catalyzes a committed step in the flavonoid pathway and uses *p*-coumaroyl-CoA as substrate to synthesize naringenin chalcone which gives the naringenin compound that accumulates in tomato fruit cuticle (Adato et al. 2009). Notably, transient silencing of chalcone synthase in tomato fruit resulted in changes in epidermal cell size and morphology (España et al. 2014a).

### Genes involved in cell wall formation

In addition to the cuticle-associated lipid pathways, the most striking gene expression changes were related to polysaccharide cell wall pathways. Three genes encoding cellulose synthases (*Solyc02g089640*, *Solyc03g097050* and *Solyc07g043390*), the catalytic moiety required for the synthesis of cellulose microfibrils, were expressed at much higher levels in the *shn2* mutant. In contrast, the expression of the *Solyc12g015770* cellulose synthase gene, which is highly expressed in developing fruit until ripening (Song et al. 2019), was reduced in *shn2*. Conversely, the expression of a COBRA-like gene (*SlCOBL1, Solyc01g065530*), which encodes a glycosylphosphatidylinositol (GPI) anchored protein regulating epidermal cell wall thickness and cellulose formation in tomato fruit (Cao et al. 2012; Niu et al. 2015), was higher in *shn2*. Interestingly, in rice, a COBRA-like protein modulates cellulose assembly by interacting with cellulose and affecting microfibril crystallinity (Liu et al. 2013). We also detected the altered expression of genes related to pectins and hemicelluloses, the matrix polysaccharides of the primary cell wall. Among the pectin-related genes were DEGs encoding three pectate lyases (*Solyc03g071570*, *Solyc09g091430* and *Solyc09g061890*), three pectinesterases (*Solyc03g123620*, *Solyc04g082140* and *Solyc09g061890*) and one pectin methylesterase inhibitor (*Solyc03g083730*) that were expressed at higher levels in the *shn2* mutant. Together with the polygalacturonases, the pectate lyases may enable the incorporation of newly synthesized cell wall components during cell expansion. Pectinesterases and pectin methylesterase inhibitor may have opposite roles in controlling the esterification status of pectins, which is thought to influence the mechanical properties of cell wall (Reca et al., 2012; Müller et al., 2013). The hemicellulose xyloglucan plays a key role in the loosening and tightening of cellulose microfibrils. The expression levels of 10 xyloglucan endotransglucosylase/hydrolase (XTH) genes were substantially higher in *shn2* mutant, with the exception of that of *Solyc07g009380,* which was reduced. XTH enzymes are involved in the integration of newly synthesized xyloglucans in the cell wall and therefore in assembly and restructuring of cell walls during cell expansion (Rose et al. 2002; Cosgrove 2005; Park and Cosgrove 2015).

Two genes encoding β-galactosidases (*Solyc06g062580* and *Solyc02g078950*), which may play roles in the modification of cell wall polysaccharides (Smith and Gross, 2000), were expressed at higher levels in the mutant while one β-glucosidase (*Solyc07g063390*) was expressed at lower level (Supplemental Table S5). In addition to the DEGs involved in the modification of cell wall polysaccharides, several DEGs encoded several proteins hypothesized to play central roles in cell expansion (Supplemental Table S5). Among them were expansins, which are important modulators of cell wall extensibility (Park and Cosgrove 2015). Three of them were expressed at much higher levels (*Solyc01g112000*, *Solyc05g007830* and *Solyc06g076220*) while one was expressed at lower level (*Solyc06g005560*). Four genes encoding arabinogalactan proteins also showed opposite patterns of relative transcript abundance in the *shn2* mutant and WT. Although the functions of arabinogalactan proteins are not well understood, they may play key roles in cell-wall architecture and composition (MacMillan et al. 2010). We also detected three DEGs encoding extensin-like proteins, which are hydroxyproline-rich glycoproteins essential for cell-wall assembly and growth by cell expansion, that were expressed at higher levels. Several other genes that may be involved in the coordination of epidermal cell wall development were also differentially expressed, including DEGs encoding EXORDIUM-like proteins (Sousa et al. 2020), Protodermal factor 1, and Long Cell-linked Cotton fiber protein (Supplemental Table S5).

### Genes involved in transcriptional regulation

As could be expected, given the large transcriptional changes induced by the *shn2* mutation and the role of *SHN* genes in transcriptional regulation, the expression of 78 transcription factors (TFs) was altered, in addition to TFs involved in hormonal signaling. MYB (5), WRKY (15), AP2/B3 (2), bHLH (3), BZIP (3), GATA (2), GRAS (5), NAC (5), C2H2 zinc finger (13), Dof (2), LOB (3) and other TF categories (20) were expressed at higher (63) or lower (15) levels in *shn2* (Supplemental Table S5). All WRKY genes, which are well-known TFs regulating plant abiotic and biotic stress tolerance (Li et al. 2020a), were expressed at higher levels in the *shn2* mutant. In addition, Dehydration-responsive element-binding (DREB) proteins, which also play a critical role in abiotic stress tolerance in plants (Lata and Prasad 2011), were expressed at much higher levels in the *shn2* mutant. To our knowledge most of these TFs have not previously been associated with TFs regulating fruit epidermis patterning or cuticle deposition (Borisjuk et al. 2014; Hen-Avivi et al. 2014), except for the MIXTA-like MYB transcription factor (Lashbrooke et al. 2015) and for HD Zip IV HDG2, a GLABRA 2-like protein that is a regulator of epidermal cell fate determination (Shi et al. 2013). Notably, a DEG encoding a chromatin structure-remodeling complex protein BSH (*Solyc11g013410*), which is a member of a multiprotein machinery controlling DNA accessibility with roles in the regulation of developmental and hormonal signaling pathways (Sarnowska et al. 2016), was expressed at much lower levels in the *shn2* mutant (Supplemental Table S6).

### Genes involved in hormonal signaling and regulation of plant development

In addition to the above-mentioned TFs, the expression of DEGs encoding TFs involved in signaling of various hormones (gibberellin, ethylene, auxin, abscisic acid, brassinosteroid, jasmonate) were altered (Table 2). Moreover, the expression of DEGs encoding enzymes involved in hormone (gibberellin, ethylene, auxin, abscisic acid, jasmonate) biosynthesis was also altered. Genes involved in ethylene biosynthetic and signaling pathways (26 DEGs) were by far the most affected. Ethylene is a central hormone that fulfils various roles in plant and fruit development, among which the coordination of fruit ripening in tomato (Fenn and Giovannoni 2021), and the response to biotic and abiotic stresses (Müller and Munné-Bosch 2015). ACC synthase (ACS) and 1-aminocyclopropane-1-carboxylate oxidase (ACO) catalyze committed steps in ethylene biosynthesis. The ethylene signaling and response pathway includes the ethylene receptor (ETR6 is down-regulated in *shn2*) and Ethylene Response Factors (ERFs), which belong to the transcription factor family APETALA2/ERF. ERF are well suited to mediate the diversity of ethylene responses and the interactions with other hormones and redox signaling (Müller and Munné-Bosch 2015; Liu et al. 2016). The expression of twenty-three (23) DEGs encoding ERFs, among which possible transcriptional activators or repressors (Liu et al. 2016), were altered in *shn2*. After ethylene, the largest categories of hormone-related DEGs were auxin (13 DEGs), gibberellins (5 DEGs) and jasmonate (5 DEGs). Auxin plays essential roles in many developmental processes among which cell wall modification during cell expansion (Majda and Robert 2018). The DEG encoding an auxin-regulated ARGOS-type protein (*Solyc12g096570*) that controls cell proliferation (Hu et al. 2003) was expressed at a much lower level. The gibberellins also regulate major aspects of plant growth and development, among which cell expansion. Interestingly, a recent study in tomato that linked a fruit firmness locus to cuticle thickness and composition (Li et al. 2020b) identified *GA2-OXIDASE* as the underlying gene. The mechanism by which GA2-oxidase, an enzyme of gibberellin catabolism that is strongly down-regulated in *shn2*, regulates cuticle formation is unknown although RNAseq analyses indicate SlSHN3 as a possible target (Li et al. 2020b). Surprisingly, relatively few DEGs were related to abscisic acid, regardless of its major role in the regulation of cuticle formation for plant resistance to pathogens and limitation of water loss (Curvers et al. 2010; Martin et al. 2017; Liang et al. 2021).

## Discussion

Considerable advances in our understanding of the formation and architecture of cuticle have been made in the recent years thanks to the use of natural or artificially-induced genetic diversity available in cultivated tomato and in related wild species (Petit et al. 2021). To better understand fruit cuticle formation, we previously screened an EMS-mutagenized mutant collection in the miniature cultivar Micro-Tom (Just et al. 2013) for *cutin-deficient* (*cd*) mutants exhibiting increased fruit glossiness (Petit et al. 2014). We next identified several causal mutations in cytochrome P450-dependent fatty acid oxidase (*SlCYP86A69)*, glycerol-3-phosphate dehydrogenase (*SlGPAT6*) and cutin synthase (*SlCUS1*) genes (Shi et al. 2013; Petit et al. 2014, 2016). Another tomato mutant collection (M82 cultivar) screened by Isaacson et al. (2009) led to the identification of mutations in *SlCYP86A69* (Shi et al. 2013), *SlCUS1* (Yeats et al. 2012) and *cd2*, a gene encoding a HD Zip IV transcription factor. These cuticle mutants proved to be precious tools to explore cuticle synthesis, structure and properties (Chatterjee et al., 2016; Philippe et al. 2016, 2020a; Moreira et al. 2020; Bento et al. 2021) and interaction with pathogens (Isaacson et al. 2009; Buxdorf et al. 2014; Fawke et al. 2019).

Cuticle formation is highly regulated throughout plant development, including during fruit growth (España et al. 2014b; Fich et al. 2016), and in response to environmental cues such as drought (Bernard and Joubès 2013). Many transcription factors (TFs) belonging to several families (AP2/ERF, DREB/CBF, MYB, HD-Zip IV and WW domain proteins) regulate wax and cutin synthesis and deposition (Borisjuk et al., 2014; Hen-Avivi et al. 2014). In tomato, exploitation of natural and artificially-induced genetic diversity led to the identification of several TFs including an HD ZipIV (*cd2*) controlling wax, cutin and flavonoid composition of fruit cuticle (Isaacson et al. 2009; Nadakuduti et al. 2012), another HD Zip IV that regulates cutin and wax content as well as trichome formation (Xiong et al. 2020) and a SlMYB12 transcription factor regulating fruit cuticle flavonoids (Adato et al. 2009). Here we show that a nonsynonymous mutation in SlSHN2, a member of the SHINE (SHN) clade of AP2/ERF regulatory TFs (Aharoni et al. 2004), affects the content and/or composition of wax, cutin, and cutin-incorporated polysaccharides, as well as the morphology of fruit epidermal cells.

An intriguing question is that of the possible redundancy of *SlSHN2* and *SlSHN3*. The two genes display similar developmental patterns and spatial localizations in epidermal cells except for the much higher expression of *SlSHN2* at 10 DPA and its restriction to the outer epidermis. However, silencing (RNAi) of *SlSHN3* (Shi et al. 2013) resulted in much milder cutin alterations than those observed in *shn2*. Although the *shn2* mutation significantly altered the expression of the two genes, the increase in *SlSHN3* transcript abundance in *shn2* is moderate, suggesting no gene compensation by *SlSHN3*. Comparison with the Arabidopsis *SHN* loss-of-function lines (Shi et al. 2011; Oshima et al. 2013) further suggests that *shn2* is a loss-of-function mutation resulting from the single non synonymous amino acid change located in a highly conserved SHN motif of SlSHN2. Because the mutation falls into the ‘mm’ domain common to the two SlSHN2 isoforms and does not alter the relative abundance of *SlSHN2*_X1 and X2 transcript variants, it probably does not affect alternative splicing of *SlSHN2*, which could play a role under adverse environmental conditions, as has been demonstrated for the tomato cuticle gene *CWP* (Chechanovsky et al. 2019).

Modifications of wax and cutin load and/or composition were amongst the most obvious biochemical alterations observed in *shn2*. The strong reduction in cutin load affected all the major fatty acids-related monomers, including the 9(10),16-dihydroxyhexadecanoic acid that was also dramatically reduced in petals of *SHN*-silenced Arabidopsis lines (Shi et al. 2011), as well as the phenylalanine-derived *p*-coumaric acid. In addition, esterification levels of 9(10),16-dihydroxyhexadecanoic acid were modified as in the *cus1* cutin synthase mutant (Philippe et al. 2016), except that the proportion of non-esterified primary OH groups were much increased and that of secondary OH groups less increased in *shn2* than in *cus1*, suggesting a different pattern of cutin cross-linking in these mutants. Another original feature of *shn2* cuticle is the striking reduction in cutin-embedded cellulose and concomitant increase in hemicellulose and pectin. Moreover, RGI branching, pectin methylesterification and acetylation of CEP were all decreased in *shn2*. Considering the increasingly obvious role of polyester-bound phenolics and CEP in cuticle features (reviewed in Philippe et al. 2020b; Reynoud et al. 2021), it is likely that changes in fruit cuticle-related properties (glossiness, permeability, firmness) and epidermal cell morphology observed in *shn2* are not only due to the reduction in cuticle thickness but also to alterations in cuticle composition and architecture (España et al. 2014b; Philippe et al. 2020a). An intriguing finding is the increased resistance of *shn2* to water-loss, which is likely independent of cutin deficiency (Isaacson et al. 2009; Philippe et al. 2016) and of the small changes in wax composition observed (Leide et al. 2007). It could be related to possible alterations in the structure of the cutin-polysaccharide-phenolic continuum, which warrant further investigations.

To date, little is known about the coordination of plant growth with the synthesis and deposition of cuticle components. Tomato mutants affected in cutin biosynthesis and assembly (Shi et al. 2013; Petit et al. 2014, 2016) or in flavonoid biosynthesis (España et al. 2014a) present variations of epidermal cell morphology reminiscent of *shn2*, suggesting feed-back regulation of cuticle deposition and epidermal patterning through a common regulatory network. Several lines of evidence, including the results presented herein, indicate that SHN TFs orchestrate cuticle formation and epidermal patterning by coordinating the synthesis of cuticle polyesters with the synthesis and modification of cuticle polysaccharides. In Arabidopsis, simultaneous silencing of the three *SHN* genes revealed that they redundantly regulate elongation and decoration of flower epidermal cells, probably through the reduction in cutin load and the modification of the cell wall pectins (Shi et al. 2011). Regulation of cuticle polysaccharides by SHN is further supported by results from ectopic expression of *AtSHN2* in rice, which oppositely increased cellulose and decreased lignin contents (Ambavaram et al. 2011). Further studies indicate that SHN and MIXTA-like regulators act coordinately to regulate cuticle deposition and epidermis differentiation. Indeed, expression of a MYB106 (a MIXTA-like TF) chimeric repressor induced cuticle deficiencies and reduced cutin nanoridges in Arabidopsis flowers (Oshima et al. 2013) i.e. phenotypes similar to those obtained by triple knockdown of *SHN* genes (Shi et al. 2011) and expression of a SHN1 chimeric repressor. In tomato, overexpression of *SlSHN1* affected leaf wax deposition and plant drought resistance (Al-Abdallat et al. 2014) while RNAi silencing of *SlSHN3* modified wax and cutin synthesis, epidermal patterning and pathogen susceptibility (Shi et al. 2013). Similar traits were affected by silencing tomato *SlMIXTA-like*, which was proposed to act downstream of *SlSHN3*, possibly with HD Zip IV TFs (Lashbrooke et al. 2015).

Consistent with the biochemical alterations observed in *shn2*, transcriptome analysis revealed a set of genes associated with cutin synthesis and assembly and with the phenylalanine-derived pathway. These genes are likely targets of SlSHN2 as their functions overlap with those of *SHN* targets previously reported in Arabidopsis (Shi et al., 2011; Oshima et al. 2013) and *SlSHN3* and *MIXTA*-like targets in tomato (Shi et al. 2013; Lashbrooke et al. 2015). Accordingly, *SlMIXTA-like*, which is also a target of *SlSHN3* (Shi et al. 2013), was down-regulated in *shn2*. In addition, the expression of numerous genes associated with cell wall synthesis and modifications were altered. Co-regulation of genes related to lipid polyesters and cuticle polysaccharides has been observed in several cutin biosynthesis mutants (Voisin et al. 2009; Petit et al. 2016), which is not surprising considering the tight interactions between cuticle components (Reynoud et al. 2021). Our results strongly suggest a central role for SlSHN2 in the control of cuticle deposition and architecture through the coordinated regulation in the growing fruit of genes associated to cutin and polysaccharide deposition. An intriguing feature is that the expression of the majority of cell wall-related genes detected is increased in *shn2* while, at the opposite, ectopic expression of *AtSHN2* in rice increased the expression of cellulose and other cell wall biosynthesis pathway genes (Ambavaram et al. 2011). The other feature highlighted by transcriptome analysis of *shn2* is the considerable number of DEGs related to phytohormones biosynthesis and signaling pathways. Our results support the role of GA in cuticle deposition, already highlighted in previous studies on Arabidopsis petal development (Shi et al. 2011) and tomato fruit firmness (Li et al. 2020b). They further position SlSHN2 in the regulation network upstream of epidermis-expressed GA 2-oxidase, which likely controls active GA levels in epidermal cells. Additionally, our findings suggest a central role for ethylene in SlSHN2 coordination of cuticle deposition, epidermal patterning and defense against biotic and abiotic stresses.

## Conclusion

Tomato fruit undergoes a considerable increase in size during the cell expansion phase (Lemaire-Chamley et al. 2005; Musseau et al. 2020), a process to which the fruit must continually adapt by adjusting the morphology of its epidermal cells and the architecture and properties of the cuticle. In this study, *SlSHN2* emerged as a key regulatory gene in the genetic program determining the adaptation of fruit epidermal and sub-epidermal cells to fruit growth. Hormonal signaling cascades and possibly epigenetic control of gene expression are likely part of the regulation network involving *SlSHN2*. Future studies positioning *SlSHN2* in the genetic network that controls early fruit development will provide insights into how cuticle component synthesis and epidermal patterning are coordinated with fruit growth processes.

## Material and methods

### Plant materials

The tomato (*Solanum lycopersicum*) *glossy* mutant line P7B8 was isolated from an EMS (ethyl methanesulfonate) mutant tomato collection generated in the miniature Micro-Tom cultivar at INRAE (Bordeaux, France), as previously described (Petit et al. 2014). Thirty M_2_/M_3_ mutant families were selected in silico by screening our phenotypic mutant database (Just et al. 2013) for fruit cracking mutants. Following observation of 12 plants per family, one of the individuals of the P7B8 family showing a glossy fruit phenotype was isolated, selfed, and a single individual of the offspring carrying the homozygous *glossy* mutation was isolated. The parental Micro-Tom line used for EMS mutagenesis (Just et al. 2013) was used as a control unless otherwise indicated. An additional control (WT-like) used for several experiments (e.g. RNA-seq analysis) was a pool of P7B8 recombinant individuals carrying the same set of mutations as the *shn2* mutant except for the *shn2* mutation. All plants were grown in greenhouse under the conditions described in Rothan et al. (2016). Fruit glossiness was measured at the mature green (MG) and Red Ripe (RR) stages; photographs were taken under standardized conditions as described in Petit et al. (2014).

### Mapping-by-sequencing

MBS and recombination analysis were performed as previously described (Garcia et al. 2016; Petit et al. 2016). A mapping BC_1_F_2_ population of 216 plants was created by crossing the P7B8 mutant with a WT parental line and by selfing a single BC_1_F_1_ hybrid. Two bulks were then constituted by pooling 38 plants displaying a glossy fruit phenotype (glossy bulk) or 38 plants with a dull/moderate glossy phenotype (WT-like bulk). DNA extracted from each bulk was used for preparation of libraries that were sequenced using a HiSeq 2500 sequencer (Illumina, 100-bp paired-end run mode) at the INRA-GeT-PlaGe-GENOTOUL platform. Sequence analyses were performed as previously described by Garcia et al. (2016), using version SL3.0 of the reference tomato genome for read mapping (ftp://ftp.solgenomics.net/genomes/Solanum_lycopersicum/Heinz1706/assembly/build_3.00/). EMS variants with a read depth between 10 and 100 were considered for allelic frequency analysis. Recombinant BC_1_F_2_ individuals were detected using the EMS-induced SNPs flanking the putative mutation as markers in a Kompetitive allele-specific PCR (KASP) assay (Smith and Maughan, 2015). Subsequent genotyping of the causal *shn2* mutation was done through Sanger sequencing of PCR products (SHNseqF1 AGCAGAAGAAGCAGCAAGAGCAT; SHNseqR1 GGGGATACTTGTGCATTATCCAA). Filtered EMS mutations were annotated using snpEff version 4.1 (Cingolani et al 2012) from build release SL3.0 of the reference tomato genome.

### SlSHN2 Phylogenetic analysis

*A. thaliana* and *S. lycopersicum* databases (TAIR 2021 and SGN 2021 respectively) were searched for SHINE amino acid sequences. Neighbor-joining (NJ) tree was then constructed using MEGA 11.0 (Tamura et al. 2021) and CLUSTALW (Larkin et al. 2007) with default parameters settings.

### Measurement of the properties of fruit cuticle

For measurements of cuticle permeability to stain, we used a protocol adapted from Tanaka et al. (2004). MG fruits collected from WT and *shn2* mutant plants were placed in 0.1% toluidine blue solution for 16 h. Staining of the fruit surface was then visually scored and photographs were taken. For water loss measurements, red ripe (RR) fruits were harvested from WT, WT-like and *shn2* plants (5 fruits from 3 plants). For fruit sealing, Patafix (UHU) was then applied on peduncles and fruits were stored at room temperature. Fruit fresh weight was recorded at time zero and each week, until 40 days. Water loss was calculated as a percentage of weight loss. Fruit firmness was assessed by penetrometry using a Fruit Texture Analyser (GüSS, South Africa) equipped with a 5-mm diameter probe as previously described (Musseau et al. 2020). A minimum of 6 fruits from 6 different plants were analyzed as biological replicates.

### Microscopy analysis of fruit cuticle

For measurement of cuticle thickness between two adjacent epidermal cells, microscopic observations were made on freshly peeled outer epidermal tissue from WT and *shn2* red ripe stage fruits and the width of the cutinized cell wall between two adjacent epidermal cells was measured as previously described (Petit et al. 2016). For the evaluation of the extent of cell cutinisation and morphology, transverse fresh sections of fruit exocarp were obtained from 3 independent 20 DPA stage fruits from WT and *shn2* plants. The sections were sequentially stained with calcofluor white stain (Sigma-Aldrich) for cell walls and BODIPY 493/503 (Thermo Fisher Scientific) for cuticle lipids. The sections were submerged for 30 s in 0.05% calcofluor white stain and washed for 5 min with PBS two times. They were then stained with BODIPY solution (1 μg BODIPY per mL of PBS) for 5 min and washed in 1 ml of PBS and washed again. The stained sections were mounted on a slide in fluorescence mounting medium (CITIFLUOR AF1, England) and observed by confocal microscopy (Zeiss LSM880, Germany). Images were acquired with Zen 2011 software. All these experiments were realized at the Bordeaux Imaging Center (http://www.bic.u-bordeaux.fr/). Observations were made on WT, *shn2* homozygous mutant (*shn2*/*shn2*) and WT-like (*SHN2*/*SHN2*) individuals from the mapping BC_1_F_2_ population detected by KASP assay. For each genotype, a minimum of three pericarp sections from three fruits from different plants grown side-by-side were observed.

### Cutin and wax analysis

Cuticular waxes were extracted by fruit immersion for 30 s in 6 mL of chloroform containing 6 µg of docosane as an internal standard and subsequently analyzed as previously described (Petit et al. 2014). For the cutin monomer analysis, two 1 cm diameter discs were isolated from a RR fruit epidermal peel, carefully scratched with a scalpel blade to remove exocarp cells and incubated for 30 min in isopropanol at 85 °C. The cutin was then delipidated, depolymerized and analyzed as previously described (Petit et al. 2014). Quantitative measurements were performed by gas chromatography with a Hewlett-Packard 5890 series II gas chromatograph equipped with a flame ionization detector (Bourdenx et al. 2011).

### Sugar analyses

Cutin samples were prepared from isolated fruit peels and treated with cellulase and pectinase, as previously described (Girard et al. 2012), followed by 70% ethanol treatment and extensive dewaxing with chloroform. Polysaccharides were hydrolyzed by a two-step hydrolysis protocol using inositol as internal standard, as previously described (Philippe et al. 2020a). The alditol acetates of noncellulosic sugars released after sulfuric acid hydrolysis (1 M, 2 h, 100 °C) were analyzed by gas chromatography. To measure cellulosic glucose, a pre-hydrolysis step was included (72% H2SO4 for 30 min at 25 °C). Alditol acetates were analyzed on a Perkin Elmer AutoSystem (Perkin Elmer, Courtaboeuf, France) mounted with an OV-225 capillary column (J&W Scientific, Folsom, CA, USA; length 30 m, internal diameter 0.32 mm, operating at 205 °C with H2 as the carrier gas). The quantification of uronic acids was performed on diluted aliquots using the meta-hydroxydiphenyl colorimetric method (Blumenkrantz & Asboe-Hansen, 1973). Cellulose content was deduced from the glucose specifically released during the pre-hydrolysis step. Hemicellulose content was deduced from the amount of total neutral sugars (except glucose) without the pre-hydrolysis step. Pectin content was evaluated from the amount of uronic acid.

After an alkaline hydrolysis, the content of methanol released from the methyl esters substituting pectins was determined with an alcohol oxidase (*P. pastoris*, Sigma-Aldrich) by the N-methylbenzothiazolinone-2-hydrazone method (Anthon and Barrett, 2004). The acetic acid released was measured with a specific enzymatic kit (K-ACET, Megazyme, Wicklow, Ireland). The degree of methyl esterification refers to the mole amount of methanol per 100 mol of uronic acid, while the degree of acetylation (that can be released from pectin and hemicelluloses) refers to the mass of acetic acid per mass of polysaccharides.

### RNAseq analysis

Twelve 1 cm diameter discs of epidermal peels were isolated from six 20 DPA fruits collected from three independent WT and *shn2* mutant plants and carefully scratched with a scalpel blade, as above. Two disks per fruit were collected in distinct pools and three pools were made in order to obtain three biological replicates. Samples were ground in liquid nitrogen and stored at -80 °C until RNA extraction. RNA was extracted using the RNA purification from plant and Fungi Nucleospin kit (Macherey–Nagel, Bethlehem, PA, USA), by following the supplier's recommendations. Total RNA integrity and concentration were assessed using an Agilent 2100 Bioanalyzer with RNA Nano Chip (Agilent Technologies, Santa Clara, California, USA). Total RNA samples were sequenced at the BGI Tech Solutions (Hong Kong SAR, China) facility. Bioinformatic analysis was performed with the integrated web-based BGI tool ‘Dr.Tom’ (https://www.bgi.com/global/dr-tom/). The Filtered RNA-seq reads were aligned to the tomato reference genome sequence *S. lycopersicum* build release SL3.00 (Species: Solanum_lycopersicum_4081; Source: NCBI; Reference Genome Version: GCF_000188115.4_SL3.0) using HiSAT. Bowtie2 was used to align the clean reads to the reference genes. Annotated transcripts were obtained using ITAG4.0 gene models. Volcano Plot representation was done using R software with the EnhancedVolcano package, using data with following specifications:-1 < log2FC < 1; FPKM > 5, q Value < 0.07. The gene ontology (GO) enrichment analysis was performed with BLAST2GO (2021), with default parameters.

### Statistical analysis

When appropriate, a Student’s *t* test was performed.

### Supplementary Information

**Additional file 1:** **Supplemental Figure S1**. Digital expression of tomato SlSHN2 and SlSHN3 genes during tomato fruit development. **Supplemental Figure S2.** Transcripts variants of SlSHN2. **Supplemental Figure S3.** Developmental, cuticular and epidermal phenotypes of WT-like plants. **Supplemental Figure S4.** Distribution of down- and up-regulated genes in 20 DPA fruit exocarp of WT‐like and shn2.

**Additional file 2:** **Supplemental Table S1.** Mapping-by-sequencing data. **Supplemental Table S2.** Wax composition of the fruit cuticles of wild-type (WT) and shn2. **Supplemental Table S3.** Cutin composition of the fruit cuticle of wild-type (WT) and shn2. **Supplemental Table S4.** DEGs involved in transcriptional regulation. **Supplemental Table S5.** DEGs with possible roles in cell wall modifications. **Supplemental Table S6. **DEGs associated with epidermal patterning and development.

## Data Availability

The lists of DEGs, log2 ratios and *q*-values for several categories of differentially expressed genes are available in Tables 1 and 2 and in Supplemental Information (Supplemental Tables S4, S5 and S6). The RNAseq data (FASTQ files) and seeds of *shn2* tomato mutant underlying this article will be shared upon request to the corresponding author.

## Abbreviations

SGN: Sol Genomics Network
TAIR: The Arabidopsis Information Resource
