# Supplementary material for: The SlSHN2 transcription factor contributes to cuticle formation and epidermal patterning in tomato fruit
Source: Mol Hortic. 2022 Jun 7;2:14. doi: 10.1186/s43897-022-00035-y (PMC10515250; doi:10.1186/s43897-022-00035-y)
Supplement: Supplementary file 1 — Additional file 1: Supplemental Figure S1. Digital expression of tomato SlSHN2 and SlSHN3 genes during tomato fruit development. Supplemental Figure S2. Transcripts variants of SlSHN2. Supplemental Figure S3. Developmental, cuticular and epidermal phenotypes of WT-like plants. Supplemental Figure S4. Distribution of down- and up-regulated genes in 20 DPA fruit exocarp of WT‐like and shn2. [file 43897_2022_35_MOESM1_ESM.pdf]

Supplemental Information

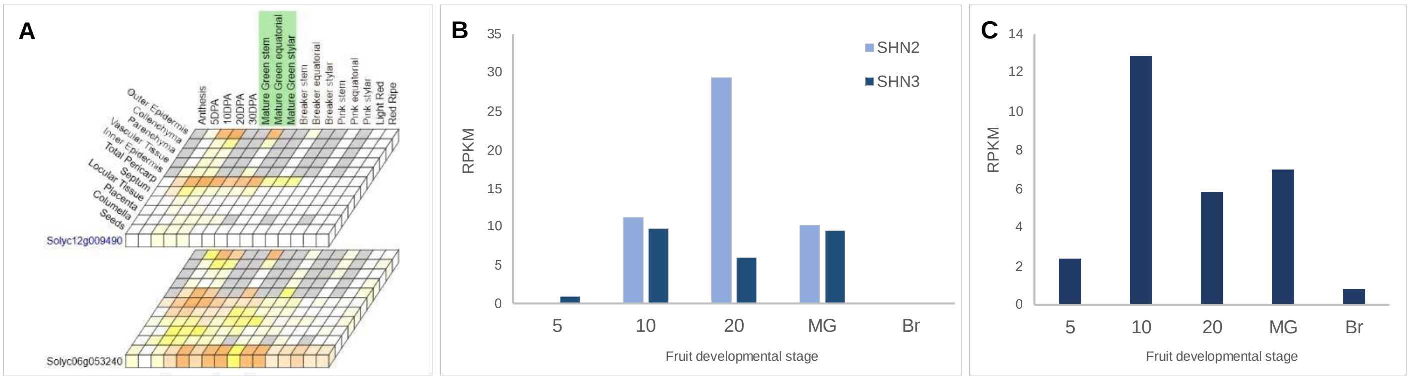

Supplemental Figure S1. Digital expression of tomato *SlSHN2* and *SlSHN3* genes during tomato fruit development. Data were retrieved from the Tomato Expression Atlas database (TEA-SGN https://tea.solgenomics.net/). A, Comparison of the expression of *SlSHN2* (*Solyc12g009490*) and *SlSHN3* (*Solyc06g053240*) in the different tissues of the fruit. B, Comparison of *SlSHN2* and *SlSHN3* expressions in the outer epidermis. C, Expression of *SlSHN3* in the inner epidermis.

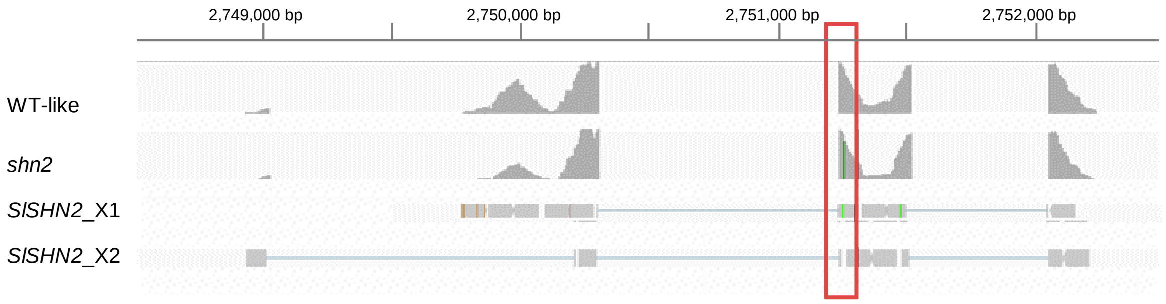

Supplemental Figure S2. Transcripts variants of *SlSHN2*.

IGV visualization of RNA-Seq analysis of 20 DPA fruit exocarp from WT-like and *shn2*. Read alignment to SL3.0 reference genome shows the presence of two transcript variants, SHN2_X1 (2 introns; XP_004251720) and SHN2_X1 (3 introns; XP_004251719) for *SlSHN2* (*Solyc12g009490*). In red box, the position of the mutation.

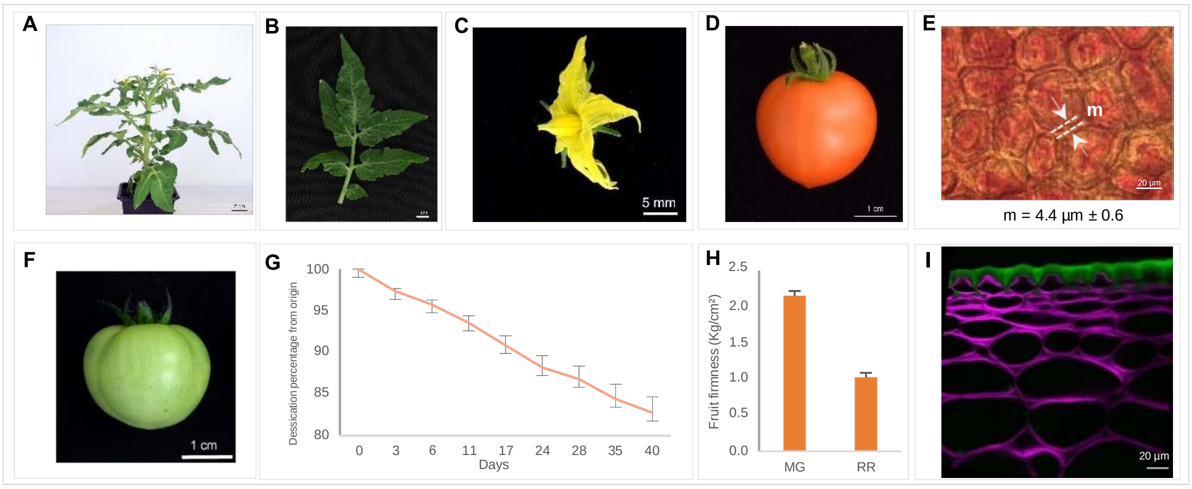

Supplemental Figure S3. Developmental, cuticular and epidermal phenotypes of WT-like plants.

A, Appearance of the plants 6 weeks after sowing. B, Morphology of the fourth leaf. C, Morphology of the flower at anthesis. D, Fruit colour and glossiness. E, Microscopic observations of freshly peeled outer epidermis of red ripe (RR) fruit. The width of the cutinizedcell wall was measured between the two white arrowheads (m). Mean values (in µm) of 100 measures (10 measures on 10 sections) are given with SD. ($P <$ 0.01 (Student's *t* test)). F, Macroscopic observations of cuticle permeability of mature green (MG) fruits after overnight soaking in 0.1% toluidine blue. G, Water loss progression in RR fruit was measured over 40 d of postharvest storage at room temperature. (n = 5). H,Fruit firmness of MG and RR fruits. Values are mean ± SD of 3 measures on 6 fruits. a, $P < 0.01$ (Student's *t* test). I, Confocal fluorescence microscopy of exocarp cell layers using Calcofluor and Bodipy dual staining showing normal cuticle deposition and conical epidermal cell shape as in WT. Scale bars are indicated on each pictures. MG, mature green; RR, red ripe.

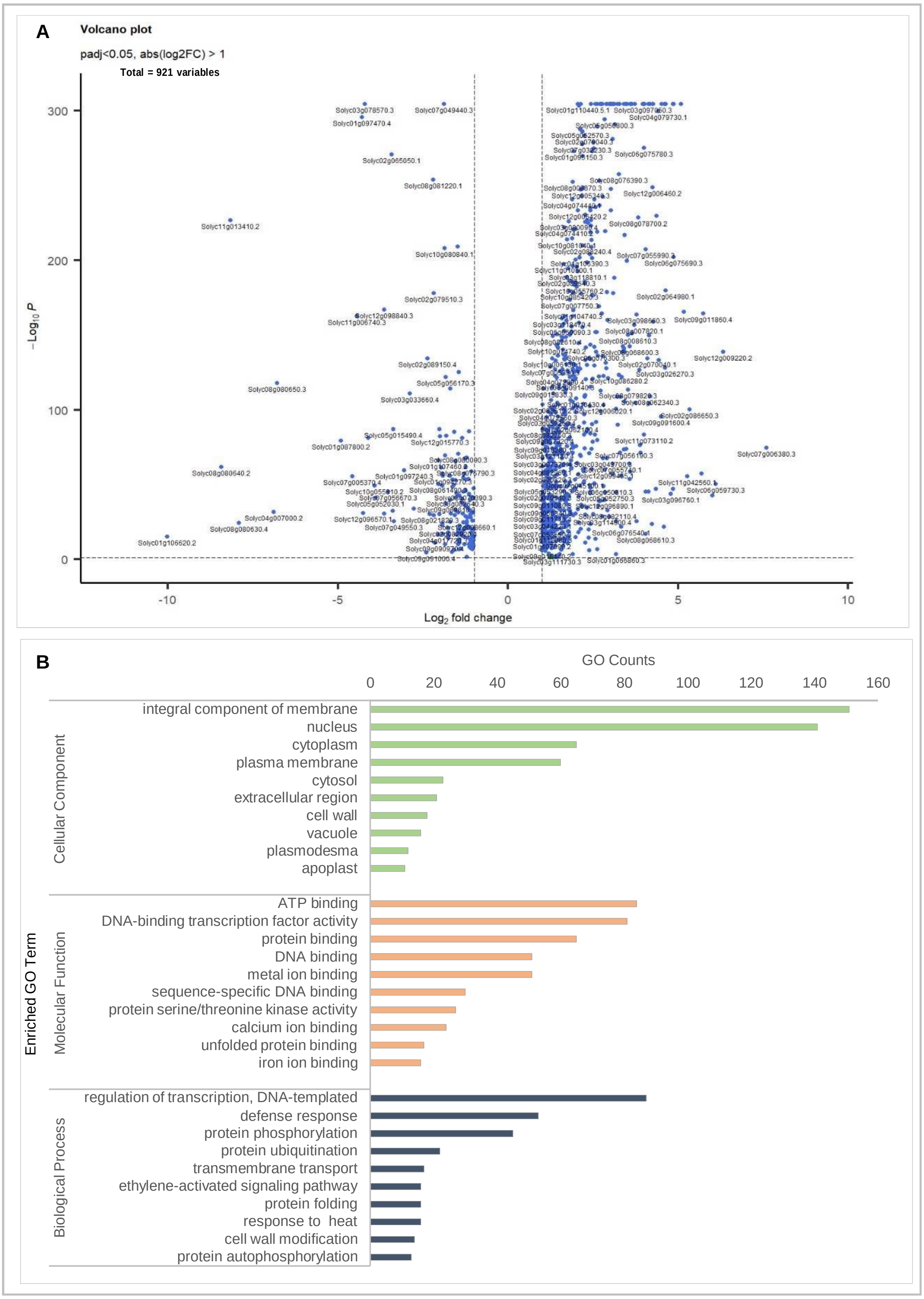

Supplemental Figure S4. Distribution of down- and up-regulated genes in 20 DPA fruit exocarp of WT-like and *shn2*. A, Volcano Plot representing 921 genes with their distribution (under-expressed, left; over-expressed genes, right). B, Top10 gene ontology terms enriched among the DEGs classified under the cellular component, molecular function and biological process categories.
