# Supplementary material for: The SlSHN2 transcription factor contributes to cuticle formation and epidermal patterning in tomato fruit
Source: Mol Hortic. 2022 Jun 7;2:14. doi: 10.1186/s43897-022-00035-y (PMC10515250; doi:10.1186/s43897-022-00035-y)
Supplement: Supplementary file 2 — Additional file 2: Supplemental Table S1. Mapping-by-sequencing data. Supplemental Table S2. Wax composition of the fruit cuticles of wild-type (WT) and shn2. Supplemental Table S3. Cutin composition of the fruit cuticle of wild-type (WT) and shn2. Supplemental Table S4. DEGs involved in transcriptional regulation. Supplemental Table S5. DEGs with possible roles in cell wall modifications. Supplemental Table S6. DEGs associated with epidermal patterning and development. [file 43897_2022_35_MOESM2_ESM.pdf]

Supplemental Information

Supplemental Table S1: Mapping-by-sequencing data.

Supplemental Table S1A

	Mutant-like bulk	WT-like bulk
Scored phenotype	glossy fruit	normal fruit
Tomato reference genome	Heinz 1706 (SL3.0)	Heinz 1706 (SL3.0)
Nb of BC_1F_2 plants per bulk	38	38
Nb of paired reads	202,404,992	209,326,654
% mapped reads	95.56%	84.73%
Insert size	280 bp	382 bp
Sequence length	150 bp	150 bp
Coverage	32X	33X

Supplemental Table S1B

Chromosome	Total variants[a]	EMS mutations[b]
0	3,473	136
1	4,100	261
2	3,512	145
3	3,500	222
4	7,714	179
5	4,240	238
6	2,316	184
7	2,749	200
8	2,717	314
9	2,618	267
10	2,837	320
11	3,690	262
12	3,167	313
all	46,633	3,041

a: total variants (natural polymorphisms and EMS mutations) obtained in the mutant and WT-like bulks (10<read depth<100)

b: EMS mutations obtained after removing natural polymorphisms between Micro-Tom and Heinz 1706 (10<read depth<100)

Supplemental Table S1C

Mutant-like bulk	WT-like bulk	No of EMS mutations
AF>0.9	0.2<AF<0.4	7
AF>0.95	0.2<AF<0.4	4

AF is the allelic frequency

Supplemental Table S1. Mapping-by-sequencing data.

A) Illumina sequencing of BC_1F_2 bulked individuals displaying a mutant highly glossy fruit or a WT normal fruit. Sequence of the reference genome is the tomato whole genome shotgun chromosomes from build release SL3.0 available on the SGN website (http://solgenomics.net). B) Number of SNPs in the mutant and the WT-like bulks for the highly glossy fruit mutant. Only variants with a read depth between 10<DP<100 were considered to remove false positive variants due to erratic read mapping. Number of total variants (natural polymorphisms between Heinz 1706 and Micro-Tom and EMS mutations) and EMS mutations per chromosome are reported in the second and third column, respectively. C) Identification of the putative causal mutations associated with the highly glossy fruit phenotype based on allelic frequency analysis in the mutant and WT-like bulks. In case of a recessive mutation (i.e. most EMS mutations), all BC_1F_2 individuals that exhibit the mutant phenotype are homozygous for the causal mutation (frequency =1 in the mutant-like bulk). On the contrary, in the WT-like bulk, the EMS mutation segregates as a mendelian locus (frequency ~0.33 in the WT-like bulk). The table reports the number of putative causal mutations corresponding to three different allelic frequency filters applied to detect the recessive causal mutation. The number of candidate causal mutations decreases with stringency of allelic frequency cut-offs. AF= Allelic Frequency.

Supplemental Table S2: Wax composition of the fruit cuticles of wild-type (WT) and *shn2.*

		Wild Type				*shn2*				
Composition	Classification	Mean	±	SD	Percentage	Mean	±	SD	Percentage	Significance
Fatty Acids	C16	1.7	±	0.2	1.9	2.6	±	0.5	2.9	
	C18	1.2	±	0	1.4	2.4	±	0.3	2.7	a
	C24	6.4	±	3.8	7.2	5.5	±	1.6	6.1	
Alkanes	C25	1	±	0.1	1.2	0.4	±	0.1	0.4	a
	C27	8.4	±	1.7	9.4	2.9	±	1	3.2	b
	C28	0.3	±	0.1	0.3	0.2	±	0.1	0.3	
	C29	5.2	±	0.7	5.9	4.6	±	1.2	5.2	
	C30	2.4	±	0.4	2.7	2.1	±	0.4	2.4	
	C31	26.9	±	2.5	30.2	28.1	±	3.7	31.3	
	C32	3.8	±	0.4	4.2	4	±	0.8	4.5	
	C33	7.1	±	0.9	7.9	11.2	±	2.3	12.5	
	C35	0.1	±	0	0.1	0.2	±	0.1	0.2	
	iso C29	0.1	±	0	0.1	0.2	±	0.1	0.2	
	iso C30	0.6	±	0.2	0.7	0.6	±	0.1	0.7	
	iso C31	2.9	±	0.2	3.2	4	±	1	4.5	
	iso C32	0.6	±	0.1	0.7	0.9	±	0.3	1	
	iso C33	0.5	±	0.1	0.6	0.7	±	0.2	0.8	
	ante iso C29	0.7	±	0.9	0.8	0.7	±	0.7	0.7	
	ante iso C31	0.5	±	0.6	0.5	0.6	±	0.7	0.7	
Amyrins	δ	0.1	±	0.1	0.2	0.2	±	0.1	0.2	
	β	1.7	±	0.3	1.9	1.4	±	1.1	1.6	
	α	3.8	±	0.4	4.3	5	±	1.8	5.5	
Lupeol		1.1	±	0.1	1.2	0.8	±	0.6	0.9	
Multiflorenol		0.1	±	0	0.1	0.2	±	0.1	0.3	
ψ Taraxasterol		0.8	±	0	0.9	0.5	±	0.2	0.5	
Taraxasterol		0.2	±	0	0.2	0.2	±	0	0.2	
Not Identified		10.8	±	1.4	12.2	9.6	±	1.7	10.7	
Total Load		88.9	±	6.5		89.9	±	16.1		

Supplemental Table S2. Wax composition of the fruit cuticles from wild-type (WT) and *shn2*. Mean values (µg/cm² x 10) of each compound are given with SD (n = 3). The percentage of total wax load is indicated for individual compounds. Letters indicate significant differences from the WT composition (Student's *t* test: b, P <0.05; and a, P <0.01).

Supplemental Table S3: Cutin composition of the fruit cuticle of wild-type (WT) and *shn2.*

Composition	Classification	Wild Type				*shn 2*				Significance
		Mean	±	SD	Percentage	Mean	±	SD	Percentage	
Fatty Acids	C16:0	1.6	±	0.1	0.1	4.4	±	1.0	1.4	b
	C18:0	1.3	±	0.5	0.1	2.3	±	0.6	0.7	
Dicarboxylic Acids	C16:0 DCA	24.6	±	0.3	1.5	2.0	±	0.3	0.7	a
	C16:0 DCA (9 or 10) OH	38.5	±	9.5	2.3	5.7	±	0.3	1.9	a
ω-Hydroxy acids	C16:0 wOH	52.8	±	1.0	3.2	8.4	±	0.4	2.8	a
	C16:0 wOH 10 oxo	14.1	±	1.2	0.8	4.3	±	0.5	1.4	a
	C18:0 wOH (9 or 10) OH	34.8	±	0.3	2.1	5.8	±	0.8	1.9	a
	C18:0 wOH (9, 10) epoxy	20.3	±	1.3	1.2	4.5	±	0.5	1.5	a
Coumaric acid		20.8	±	0.7	1.2	2.0	±	0.3	0.7	a
Polyhydroxy acids	C16:0 (9/10, 16) diOH	1287.5	±	29.6	76.9	212.4	±	5.2	70.2	a
	C18:0 (9, 10, 18) triOH	9.0	±	0.7	0.5	5.5	±	0.9	1.8	b
Not identified		168.5	±	10.0	10.1	45.4	±	2.7	15.0	a
Total Load		1673.7	±	27.6		302.5	±	2.4		a

Supplemental Table S3. Cutin composition of the fruit cuticle of wild-type (WT) and *shn2*. Mean values (μg/cm²) of each compound are given with SD (n = 3). The percentage of total cutin load is indicated for individual compounds. Letters indicate significant differences from the WT composition (Student's *t* test: b, $P < 0.05$; and a, $P < 0.01$).

Supplemental Table S4. Differentially expressed genes (DEGs) involved in transcriptional regulation.

Category	Gene Identification	Putative Function	Log2 Fold Change *shn2*/WT	*q* value
**MYB**	Solyc08g076010	MYB transcription factor APL	2.204356863	5.97767E-61
	Solyc02g088190	MYB transcription factor SlMIXTA-like	-1.082352226	1.00816E-59
	Solyc06g071690	R2R3MYB transcription factor 50	-1.717080934	1.61253E-56
	Solyc04g079360	R2R3MYB transcription factor 77	2.259246726	5.24037E-75
	Solyc07g053230	R2R3MYB transcription factor 83	4.848045	0
**WRKY**	Solyc04g078550	WRKY transcription factor 07	1.165441642	9.04462E-81
	Solyc12g096350	WRKY transcription factor 10	1.438977818	6.30445E-21
	Solyc08g006320	WRKY transcription factor 11	1.457307267	1.5511E-105
	Solyc01g095100	WRKY transcription factor 22	1.313519457	5.04414E-25
	Solyc01g079260	WRKY transcription factor 23	1.3444715	4.97515E-22
	Solyc10g011910	WRKY transcription factor 25	2.795560724	1.6798E-136
	Solyc06g066370	WRKY transcription factor 31	3.073523725	7.2237E-282
	Solyc09g014990	WRKY transcription factor 33	2.568002024	2.53381E-14
	Solyc03g116890	WRKY transcription factor 39	4.370831847	1.6594E-230
	Solyc01g095630	WRKY transcription factor 41	3.22635873	0
	Solyc10g009550	WRKY transcription factor 42	2.696258055	6.3165E-254
	Solyc08g008280	WRKY transcription factor 53	4.429765953	5.0401E-134
	Solyc08g082110	WRKY transcription factor 54	2.918483356	1.23607E-33
	Solyc03g095770	WRKY transcription factor 80	1.915765156	2.1872E-131
	Solyc09g015770	WRKY transcription factor 81	1.311016678	5.9639E-89
**AP2/B3**	Solyc05g009790	AP2/B3 transcription factor family protein	1.755165263	8.45438E-24
	Solyc04g007000	AP2/B3 transcription factor family protein	-6.884664036	2.33647E-32
**bHLH**	Solyc03g116340	bHLH transcription factor 025	1.59953897	8.4918E-125
	Solyc03g113560	bHLH transcription factor 079	1.280626239	1.17726E-25
	Solyc07g018010	bHLH transcription factor 139	1.235743114	1.28408E-17
**BZIP**	Solyc02g084860	Basic-leucine zipper BZIP transcription factor	1.162462075	1.43392E-27
	Solyc01g110480	Basic-leucine zipper BZIP transcription factor	1.467269942	9.0949E-137
	Solyc03g031760	Homeobox-leucine zipper protein HDG2-like	1.627980443	2.64317E-46
**GATA**	Solyc03g033660	GATA transcription factor	-2.880127122	1.314E-111
	Solyc04g015360	GATA transcription factor	1.745463183	4.153E-127
**GRAS**	Solyc11g012510	GRAS1	3.975656699	0
	Solyc01g100200	GRAS4	1.386992852	7.87138E-61
	Solyc04g064550	GRAS6	1.124626297	3.55758E-36
	Solyc06g076280	Transcription factor GRAS	1.412345591	2.93764E-63
	Solyc12g005340	Transcription factor GRAS	2.144138877	7.6945E-248
**NAC**	Solyc04g009440	NAC domain protein AY498713	1.671785461	2.68786E-59
	Solyc02g088180	NAC domain-containing protein	-2.763619156	8.58421E-35
	Solyc10g055760	NAC domain-containing protein 02	1.960225494	3.3014E-184
	Solyc06g073050	NAC domain-containing protein 14	2.47754035	7.9228E-274
	Solyc03g080090	NAC domain-containing protein 86	1.78500695	7.0133E-227
**Zinc Finger**	Solyc12g056950	Zinc finger 6 FCS-Like	-1.069577619	3.58045E-21
	Solyc08g079700	Zinc finger A20/AN1 domain stress-associated protein	1.490782324	4.2492E-104
	Solyc02g087210	Zinc finger AN1 domain stress-associated protein 12	2.464752552	2.5515E-214
	Solyc08g077060	Zinc finger LSD1-type	1.189362237	2.99265E-55
	Solyc01g107430	Zinc finger protein	-1.24898798	1.18311E-46
	Solyc01g107170	Zinc finger protein	1.438849042	9.61257E-71
	Solyc11g073075	Zinc finger protein	3.403941169	0
	Solyc06g075780	Zinc finger protein	3.989888133	7.8443E-276
	Solyc04g064770	Zinc finger transcription factor 34	-1.672724168	3.37926E-31
	Solyc05g052570	Zinc finger transcription factor 39	2.115064019	3.1518E-288
	Solyc10g008880	Zinc finger transcription factor 56	1.632048202	4.7013E-158
	Solyc11g073060	Zinc finger, C2H2	3.175549352	3.1708E-101
	Solyc12g088390	Zinc-finger protein	1.096052053	4.82183E-22
	Solyc06g062520	Dof zinc finger protein	-1.923646703	4.9763E-50
	Solyc03g121400	Dof zinc finger protein 14	2.887623944	2.5544E-128
**LOB**	Solyc02g085910	LOB domain-containing protein	-1.911082636	7.0395E-18
	Solyc03g095940	LOB domain-containing protein 21	-1.078385069	3.93279E-31
	Solyc02g092550	LOB domain-containing protein 38	-1.466702272	1.26818E-19
**Miscellaneous**	Solyc01g095460	G-box binding factor 3	1.224102308	1.15903E-65
	Solyc03g019710	MADS-box transcription Factor LETDR8	-1.151421388	1.01407E-10
	Solyc03g116320	TCP transcription factor 16	-1.441472636	3.73103E-21
	Solyc12g008800	Transcription factor DIVARICATA	1.092413855	3.9625E-08
	Solyc03g121660	Zinc finger, C2H2-type Protein indeterminate-domain 7	1.243466589	6.36093E-82
	Solyc07g063940	SCARECROW	2.410515411	8.3297E-205
	Solyc05g054170	Scarecrow-like protein 5	2.422129038	1.5362E-219
	Solyc01g090730	Squamosa promoter binding protein 08 b	1.839254296	2.7301E-107
	Solyc05g015840	Squamosa promoter binding protein 13	1.174500745	6.50529E-19
	Solyc03g111710	BTB/POZ and TAZ domain-containing protein 2	2.156611232	6.5982E-102
	Solyc02g092460	BTB/POZ and TAZ domain-containing protein 4	1.079500412	2.31231E-24
	Solyc12g007070	Heat shock transcription factor	-1.206614019	1.02072E-21
	Solyc09g065660	Heat shock transcription factor	3.740579286	2.2589E-105
	Solyc02g090820	Heat stress transcription factor B-1	1.023849306	3.1081E-58
	Solyc08g080540	Heat stress transcription factor B-2b	2.327089692	1.4188E-130
	Solyc03g026280	CRT binding factor 2	2.028289319	5.3796E-183
	Solyc03g026270	CRT binding factor 3	4.620996989	5.4058E-129
	Solyc08g007830	Dehydration-responsive element-binding protein 1E	4.856790242	1.1635E-47
	Solyc08g007820	Dehydration-responsive element-binding protein 1E	3.716717943	1.8321E-157
	Solyc03g124110	Dehydration-responsive element-binding transcription factor	2.579662838	1.5043E-152

Exocarp of WT-like and *shn2* 20 DPA fruit was analyzed. Genes were assigned manually to functional categories. Annotations are from SGN (https://solgenomics.net/).

Supplemental Table S5. Differentially expressed genes (DEGs) with possible roles in cell wall modifications

Gene Identification	Putative Function	Log2 Fold Change *shn2* / WT	q value
Solyc06g005560	Expansin 9	-1.30	6.4E-16
Solyc05g007830	Expansin 12	1.67	4.7E-28
Solyc01g112000	Expansin-like protein precursor 1	1.71	2.9E-160
Solyc06g076220	Expansin18	3.34	1.2E-122
Solyc05g010080	Lysine-rich arabinogalactan protein 19	-1.21	3.2E-25
Solyc04g074730	Classical arabinogalactan protein 5	1.06	1.1E-33
Solyc01g107340	Classical arabinogalactan protein 9	1.29	2.3E-59
Solyc08g078020	Methionine rich arabinogalactan	1.98	2.3E-15
Solyc02g078040	Pistil-specific extensin-like protein	1.48	3.0E-82
Solyc02g089250	Pollen Ole e 1 allergen/extensin	1.58	3.2E-68
Solyc01g006400	Pistil extensin like protein AGP	1.75	3.9E-37
Solyc02g090110	WAK-like kinase	1.53	2.3E-35
Solyc09g015040	Methyltransferase	1.24	4.1E-79
Solyc07g063390	β-glucosidase 16	-1.16	1.1E-34
Solyc06g062580	β-galactosidase	1.29	3.6E-39
Solyc02g078950	β-galactosidase	1.62	1.1E-97
Solyc02g084210	GDP-mannose 4,6-dehydratase	2.86	4.9E-220
Solyc07g049370	Glucan endo-1,3-beta-glucosidase 3	1.12	2.5E-60
Solyc07g005330	Glucan endo-1,3-beta-glucosidase	1.25	2.0E-129
Solyc11g068440	Glucan endo-1,3-beta-glucosidase 11	1.31	1.1E-115

Exocarp of WT-like and *shn2* 20 DPA fruit was analyzed. Genes were assigned manually to functional categories. Annotations are from SGN (https://solgenomics.net/).

Supplemental Table S6. Differentially expressed genes (DEGs) associated with epidermal patterning and development.

Category	Gene Identification	Putative Function	Log2 Fold Change *shn2* / WT	*q* value
**Epidermal Differentiation**	Solyc12g011010	Protodermal factor 1	4.63	0.0E+00
	Solyc06g034390	Protein EXORDIUM-like 7	1.33	4.3E-19
	Solyc04g074410	Protein EXORDIUM-like 1	1.65	1.0E-222
	Solyc04g074440	Protein EXORDIUM-like 1	1.89	1.5E-241
	Solyc04g074450	Protein EXORDIUM-like 1	2.09	3.6E-304
	Solyc01g079660	Long Cell-linked Cotton fiber protein	3.73	0.0E+00
	Solyc01g079670	Long Cell-linked Cotton fiber protein	4.14	4.9E-46
	Solyc11g013410	Chromatin structure-remodeling complex protein BSH	-8.16	2.2E-227

Exocarp of WT-like and *shn2* 20 DPA fruit was analyzed. Genes were assigned manually to functional categories. Annotations are from SGN (https://solgenomics.net/).
